# Monitoring and behavior of unsaturated volcanic pyroclastic in the Metropolitan Area of San Salvador, El Salvador

**DOI:** 10.1186/s40064-016-2149-x

**Published:** 2016-04-27

**Authors:** José Alexander Chávez, José Landaverde, Reynaldo López Landaverde, Václav Tejnecký

**Affiliations:** Department of Geotechnics, Faculty of Civil Engineering, Czech Technical University in Prague, Thákurova 7, 166 29 Prague 6, Czech Republic; Oficina de Planificación del Área Metropolitana de San Salvador (OPAMSS), Diagonal San Carlos 15, Avenida Norte y 25 Calle Poniente Col. Layco, San Salvador, El Salvador; Departamento de Geotecnia, Facultad de Ingeniería y Arquitectura, Universidad de El Salvador, Ciudad Universitaria, Final Av. Héroes y Mártires del 30 de Julio, San Salvador, El Salvador; Facultad de Ciencias Agronómicas, Universidad de El Salvador, Ciudad Universitaria, Final Av. Héroes y Mártires del 30 de Julio, San Salvador, El Salvador; Department of Soil Science and Soil Protection, Faculty of Agrobiology, Food and Natural Resources, Czech University of Life Sciences Prague, Kamýcká 129, 165 21 Prague 6, Czech Republic

**Keywords:** Unsaturated, Tierra Blanca Joven, Pyroclastic, Suction, Soil moisture sensor, Flowslide, Landslide

## Abstract

Field monitoring and laboratory results are presented for an unsaturated volcanic pyroclastic. The pyroclastic belongs to the latest plinian eruption of the Ilopango Caldera in the Metropolitan Area of San Salvador, and is constantly affected by intense erosion, collapse, slab failure, sand/silt/debris flowslide and debris avalanche during the rainy season or earthquakes. Being the flowslides more common but with smaller volume. During the research, preliminary results of rain threshold were obtained of flowslides, this was recorded with the TMS3 (a moisture sensor device using time domain transmission) installed in some slopes. TMS3 has been used before in biology, ecology and soil sciences, and for the first time was used for engineering geology in this research. This device uses electromagnetic waves to obtain moisture content of the soil and a calibration curve is necessary. With the behavior observed during this project is possible to conclude that not only climatic factors as rain quantity, temperature and evaporation are important into landslide susceptibility but also information of suction–moisture content, seepage, topography, weathering, ground deformation, vibrations, cracks, vegetation/roots and the presence of crust covering the surface are necessary to research in each site. Results of the field monitoring indicates that the presence of biological soil crusts a complex mosaic of soil, green algae, lichens, mosses, micro-fungi, cyanobacteria and other bacteria covering the slopes surface can protect somehow the steep slopes reducing the runoff process and mass wasting processes. The results obtained during the assessment will help explaining the mass wasting problems occurring in some pyroclastic soils and its possible use in mitigation works and early warning system.

## Background

Since Terzaghi in 1936 explained the behavior of saturated soils and helped to underline the principles of the soil mechanics, different authors (Fredlund 1997) have tried to explain the behavior of the “problematic” unsaturated soils (Fredlund et al. 2012) that didn’t fit the behavior explained by the Terzaghi’s equation. These types of soils comprise soils above the water table (sands, silts and clays and even gravels) including man-made fills, colluvium, residual soils, air transported soils and gas generating soils (Fredlund et al. 2012).

Some clays are known for changing their volume (swell or shrink) according to the water content and mineralogy (Craig 2004). Other soils are known as “collapsible” since they naturally are stiff and have low density (Houston and Houston 1997), but will collapse (losing an apparent cohesion) after saturation. Loess are well-known problematic soils (air transported) in China, Europe and other countries (Cheng et al. 2008; Xu et al. 2012, 2013; Wang et al. 2014). But also pyroclastic deposits have been reported as problematic soils also (Rolo et al. 2004; Pagano et al. 2010; Cascini et al. 2013). This situation makes that the slopes of the pyroclastics deposits are almost vertical and temporally stable; but will collapse when saturated.

In the beginning of the soil mechanics, the research was concentrated more on saturated soils since it was made in areas with a fresh or wet climate (Fredlund 1997) where the vadose zone is near to the surface. Additionally, for unsaturated soils more time was needed to develop the appropriate technology to measure the parameters and understand its behavior in laboratory and field. The urban growth throughout the world, in locations with unsaturated soils and its severe problems compelled to find practical and economical solutions. In the early 70s and 80s it was reported the loss of billions of dollars in damage to homes, buildings, roads, etc. in the United States and this type of soil was called the “hidden disaster” (Fredlund and Rahardjo 1993).

Nowadays this “problematic unsaturated soils” described before, that are in a metastable state and swell or collapse according to the moisture content, are studied into the frame of unsaturated soil mechanics. Ordinarily, the two independent stress variables used (Fredlund and Rahardjo 1993; Ng and Menzies 2007) are the net stress σ − u_a_ and matric suction u_a_ − u_w_. The term matric suction (Fredlund et al. 2012) is used to indicate the negative pressure of water relative to atmospheric air pressure. Being total stress σ, pore water pressure u_w_ and pore air pressure u_a_. But also the osmotic suction can be important in some soils (Fredlund et al. 2012). The negative pore water pressure play a key role into the behavior of these soils and the importance in engineering structures (Murray and Sivakumar 2010).

**Fig. 1:**
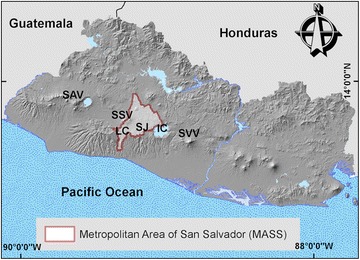
Location and topography of El Salvador and the Metropolitan Area of San Salvador. *SAV* Santa Ana Volcano, *SSV* San Salvador Volcano, *LC* Las Colinas, *SJ* San Jacinto, *IC* Ilopango Caldera, *SVV* San Vicente Volcano

The studied area for this project is located in the Metropolitan Area of San Salvador (MASS) (El Salvador, Central America), along the Ring of Fire where the subduction process causes high volcanic and earthquake activity (Fig. 1). In addition to these, it’s an area affected by hurricanes, storms and tropical depressions coming from the Pacific and Atlantic Oceans (Chavez et al. 2014). El Salvador is a low income country with poor geological sciences knowledge (Rose et al. 2004; Gonzalez et al. 2004); for this reason using low cost (but reliable) equipment will help in decrease the social, environmental and economic losses every time the country is affected by geological hazards.

Like other cities, the MASS urban growth was not planned according to the geology and the properties of the rocks and soils, but for economic and social needs (Schmidt-Thomé 1975). Rural poverty, the civil war in the 80s, overpopulation and uncontrolled urbanization (Bommer and Rodriguez 2002) induced that most of people with fragile economic resources live in risky areas (edge or inside the ravines, close to scarps, problematic soils, etc.) increasing the vulnerability to geological hazards.

**Fig. 2:**
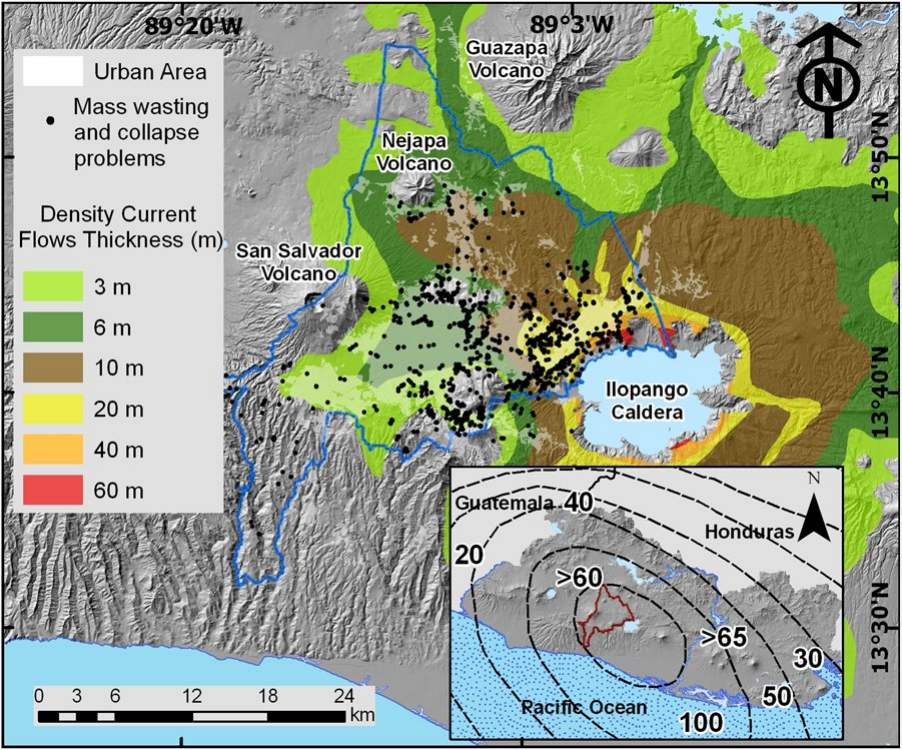
Figure only shows density current flows thickness (pyroclastic flows and surges). Most of the points of mass wasting and collapse belong to urbanized areas in thick Tierra Blanca Joven (TBJ) pyroclastics. The *inset* displays the pyroclastics thickness in cm for all the country Modified from Chavez et al. (2012)

**Fig. 3:**
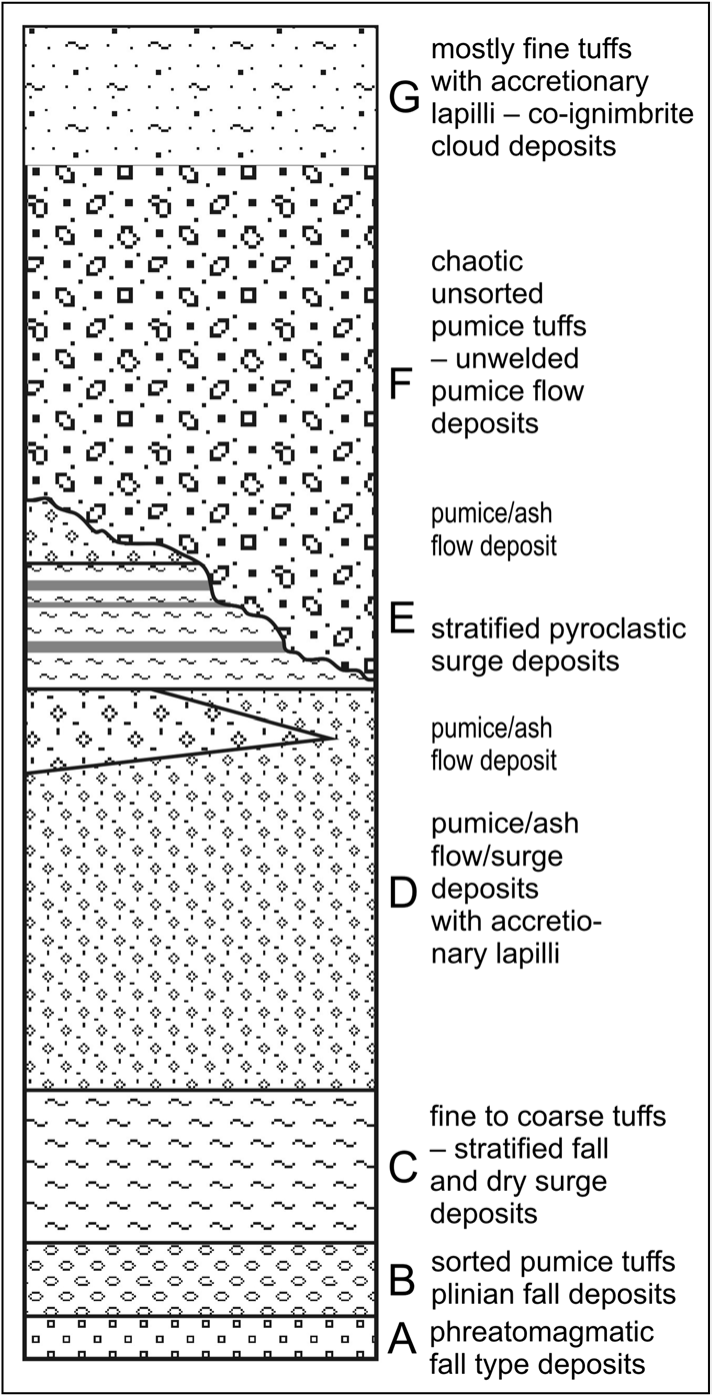
Units subdivision of Tierra Blanca Joven (TBJ) pyroclastics (Lexa et al. 2011)

Tierra Blanca Joven (TBJ), the subject of this research, is a volcanic pyroclastic that covers most of the Metropolitan Area of San Salvador (MASS), being sensitive to moisture changes and vibrations leading to intensive mass wasting processes (Fig. 2). TBJ is the last plinian eruption of Ilopango caldera deposits (belonging to the late Pleistocene to Holocene age). The TBJ deposits (poorly consolidated) are comprised (Fig. 3) of ash falls, density current flows (pyroclastic flows, surges) phreatomagmatic, coluvial and alluvial deposits (Hernandez 2004).

Erosion, landslides, liquefaction, seismic amplification, collapse and settlements have been reported in TBJ (Lomnitz and Schulz 1966; Schmidt-Thomé 1975; Hernandez 2004; Rolo et al. 2004) linked to earthquakes, rains and anthropogenic changes (housing projects, filling of ravines, cutting of slopes, irrigation, occupation of floodplains and alluvial cones as well as modification of natural drainage processes).

Authors like Amaya and Hayem (2000), Hernandez (2004), Rolo et al. (2004) and Chavez et al. (2012, 2013) have reported some of the properties and behavior of TBJ concluding that is an unsaturated collapsible soil, whose “apparent cohesion” (suction) decrease when is saturated. Usually the slopes of Tierra Blanca Joven (TBJ) are vertical (70°–90°) and can reach tens of meters.

**Fig. 4:**
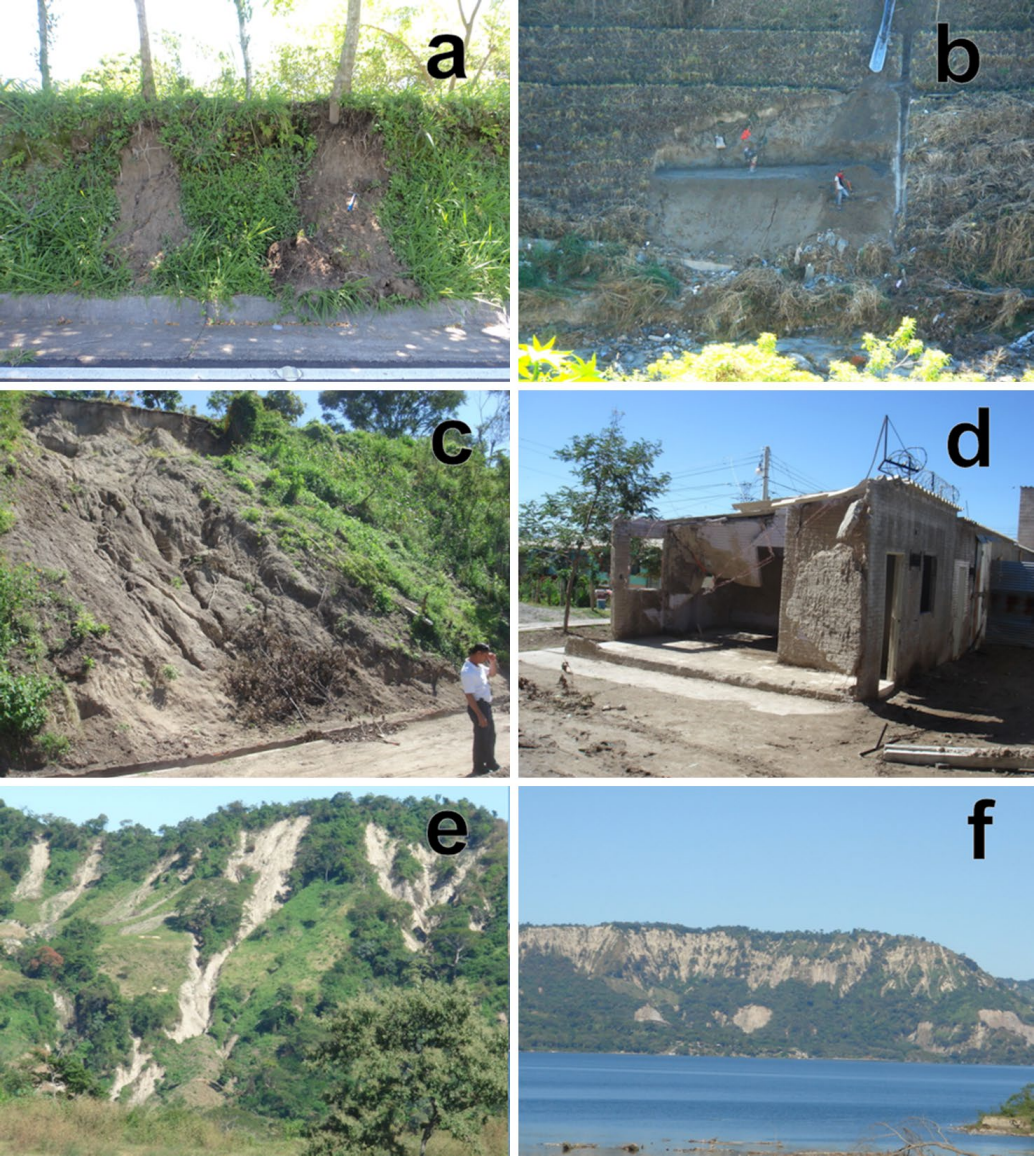
Sand/silt/debris flowslide and debris avalanche in TBJ after rains. Photo **a** are flowslides that were connected with a flat surface on top, which allowed infiltration and runoff. **b** A typical mitigation work for a TBJ slope with a flat surface on top, including drainage system and grass cover. If not regularly maintenance is made failure happens. **c**, **d** Photos belong to the same location and is a typical example of damage connected with a flowslide during Hurricane Ida in 2009 (photo **c**); that killed 3 persons inside the house in photo **d**. Photos **e**, **f** debris flowslides/debris avalanches in the hillsides of Ilopango Caldera (Hurricane Ida 2009)

Like Loess (Wang et al. 2014) TBJ pyroclastics can move as an undisturbed block of material (a combination between fall and of topple), whose failure plain is connected to the root system, thermal changes, vibrations, water acting on the cracks and loss of suction. Additionally, TBJ can also behave [definitions by Hungr et al. (2013) will be used in this paper] like a sand/silt/debris flowslide and debris avalanche (Hernandez 2004; Rolo et al. 2004; Chavez et al. 2012, 2014) (Fig. 4); this is common during the rainy season and earthquakes.

**Fig. 5:**
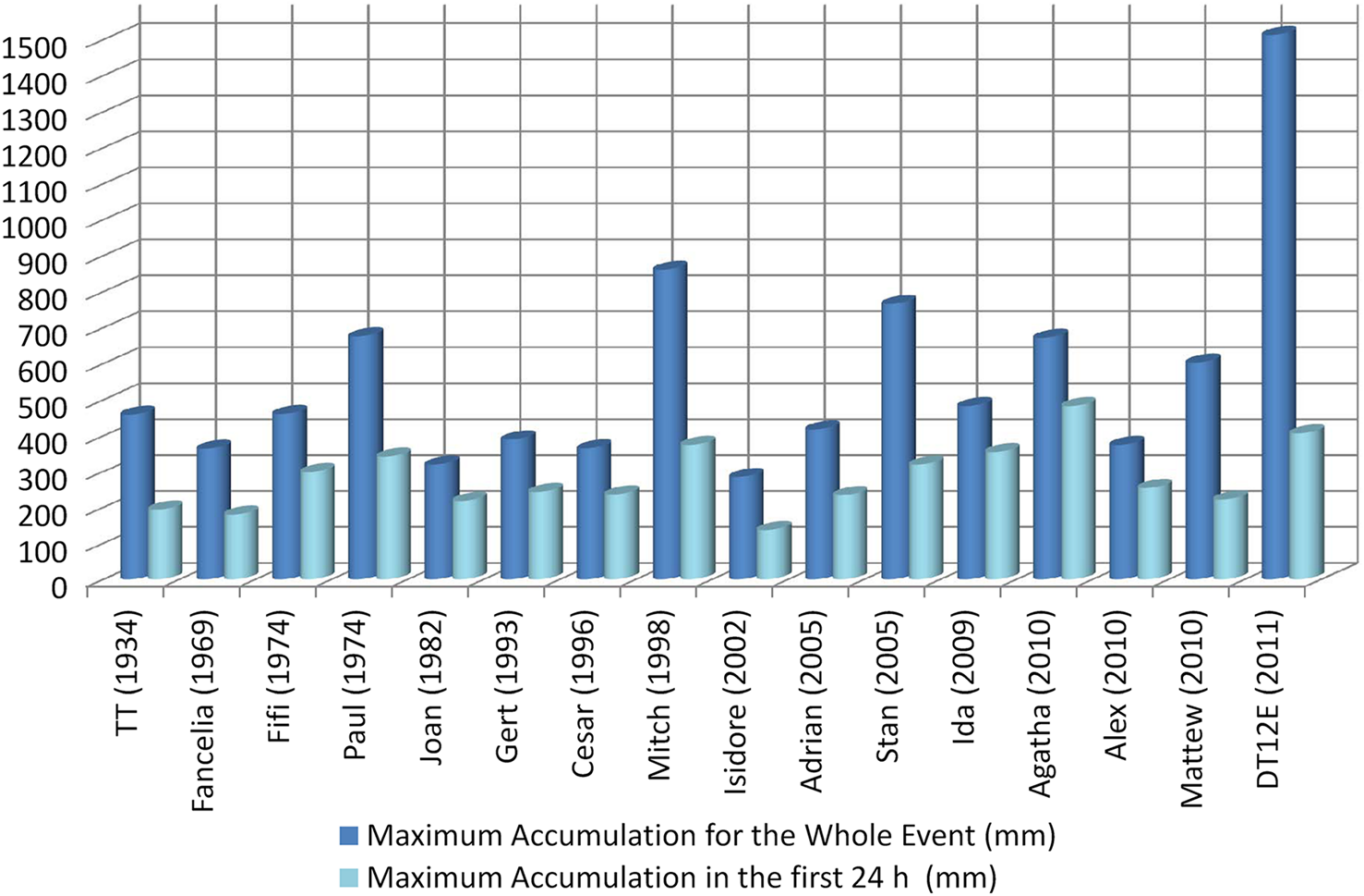
Historic comparison of important storms, hurricanes and its maximum accumulation in mm (Ministerio de Medio Ambiente y Recursos Naturales 2012)

Rains of the past years have increased mass wasting problems in the MASS, especially in areas with significant thickness of TBJ. Most of the rains associated with the initiation of extensive sand/silt/debris flowslide and debris avalanche (Ministerio de Medio ambiente y Recursos Naturales 2005) have a 24 h accumulated rain >100–200 mm and for the whole event >400 mm (Fig. 5). Similar results are reported by Pagano et al. (2010) and Wieczorek and Thomas (2010). For Cascini et al. (2013) a 24 h accumulated rain >50 mm and for the whole event >500 mm are prone to initiate a flow. For other authors the landslide-rain threshold are 78 mm/h (Ochiai et al. 2004); 66–138 mm/h (Montrasio and Valentino 2007); 100 mm/h (Moriwaki et al. 2004) and 80 mm/h (Uchimura et al. 2010).

**Fig. 6:**
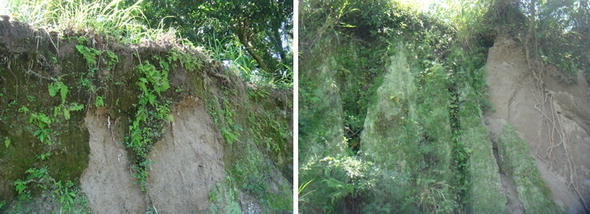
TBJ slopes with the presence of biological soil crusts (BSCs) that protect on some level against mass wasting

Field monitoring combined with laboratory tests is normally done for designing cover systems (acting as capillary barriers), foundations and for slope stability (Abdolahzadeh et al. 2011; Ng and Menzies 2007). This information is important to propose designs methods that will be reliable and can help to validate the available models. The behavior of the soil moisture during rainfall and its relationship with superficial landslides in unsaturated soils (most of them occur above the groundwater level) has raised interest in several authors as Moriwaki et al. (2004), Montrasio and Valentino (2007), Ng et al. (2008), Pagano et al. (2010), Leung and Ng (2013) and Wang et al. (2014). These authors conclude that factors associated with initiation of landslides are the decrease of suction (leading to an increase of pore pressure), seepage forces, liquefaction, permeability, lithology, presence of soil crust, ground deformation, topography, anthropic actions, intensity of rainfall events and shorter return periods, vegetation, erosion and the net normal stress (which is frequently ignored). Furthermore, the presence of biological soil crusts (BSCs) (Xiao et al. 2011) a complex mosaic of soil, green algae, lichens, mosses, micro-fungi, cyanobacteria and other bacteria can protect somehow the steep slopes reducing the runoff process and erosion (Fig. 6).

As the gravimetric/volumetric water content is a destructive method and needs time (2 days minimum) to obtain results, other methods are usually applied to obtain suction and moisture content of the soils on an ongoing basis. A direct measure of the matric suction using the Quickdraw tensiometer in the field can be useful (Pagano et al. 2010) to identify in a slope (or slopes nearby) the potential to failure, which don’t require a calibration for soil type, salinity (osmotic suction) or temperature.

Nowadays for in situ moisture content monitoring, the dielectric methods are gaining acceptance, as they can measure a wide range of moisture content and are nondestructive real time method (Qu et al. 2013; Will and Rolfes 2013). This devices help to recognize the dielectric constant of the soil, which is linked to the moisture content in the soil (Blonquist Jr et al. 2005; Kim et al. 2008).

Some dielectric methods using similar principles are the time-domain reflectometry (TDR), the frequency domain reflectometry (FDR), amplitude domain reflectometry (ADR), time domain transmission (TDT), capacitance probes and frequency domain reflectometry with vector network analyzer (FDR-V) systems (Blonquist Jr et al. 2005; Kim et al. 2008; Tarantino et al. 2008).

**Fig. 7:**
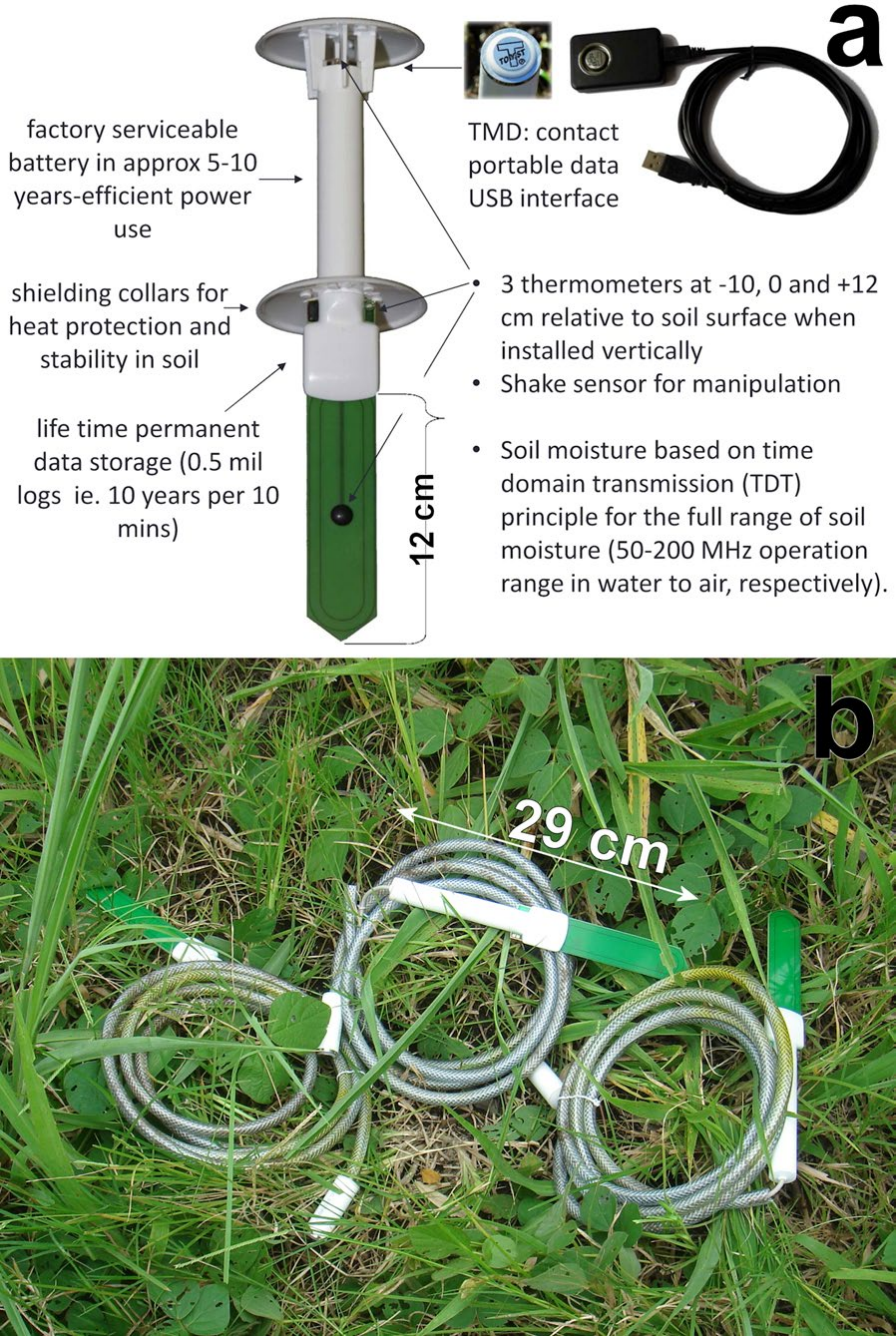
TMS3 description of components

Some of the benefits of the TDT are the reduced prize and size; also are not affected from multiple reflections, as all multiple reflections are received at later time steps than the measuring signal (Will and Rolfes 2014). TDT measurements offer advantages for investigation of inhomogeneous materials (Will and Rolfes 2013). Most of the TDT sensors consist of a two-wire line or ring oscillator that is U-shape bended allowing (Qu et al. 2013) that all the electronics are integrated in the head of the probe. Some of the TDT waveguides like SPADE and TMS3 (Qu et al. 2013) are confined within an epoxy molding material (Fig. 7) to allow inserting it into the soil. For this reason a calibration is needed to relate the values of the sensor with the actual moisture content (the device could disturb the soil matrix as mechanically efforts are required). The obtained TDT value is an average value along its length (Will and Gerding 2009).

This project attempt to research the geotechnical behavior of a pyroclastic soil (TBJ) in the field and laboratory, evaluating moisture content and suction with unsaturated soil mechanics and field monitoring using tensiometer and a Time Domain Transmission (TDT) device. For this type of material is more usual small flowslides with high density in the territory, but most of the existing research is aimed to debris flow processes. This survey will help to understand the reasons of mass wasting problems of this type of soils and its possible use in mitigation works and in early warning system. Such information can at least temporarily replace structural slope stabilization measures if the problematic is identified properly. An important aspect to research are the conditions or changes that control the failure or denudation process in the slopes and what is the key aspects in the soil that control them: suction/moisture content, cementation, weathering, evaporation, external activities or vibrations.

## Methods

Experiments, field monitoring and field observation were carried out to understand the behavior and problematic presented by the pyroclastic. The use of the Soil Water Retention curve (SWRC) within the unsaturated soil framework was necessary to include the change of suction/moisture and its connection into the pyroclastic behavior. The Quickdraw tensiometer and a TDT sensor (TMS3 data loggers of TOMST) were used for monitoring changes in suction and moisture content in TBJ volcanic pyroclastics. It was evident in the field, that the presence of a surface crust on the slopes (BSCs) and external factors control the stability of the slopes. To study the influence of areas with/without BSCs in the moisture content, some TMS3 were mounted in a slope. Installation and continuous monitoring with TMS3 devices in flowslide areas helped to understand the influence of topography in denudation, along the effect of vegetation and roots, temperature and soil use. After identifying in the slopes that the susceptibility of flowslides were connected to external factors, more TMS3 devices were installed in one monitored location to have a clearer image of the behavior of the pyroclastic. Undisturbed and disturbed samples were used during the research for TMS3 calibration curves (and to identify differences and convenience). Comparison of rain and field monitoring helped to obtain a preliminary rain threshold for the initiation of flowslides. H/L measurement of monitored flowslides were collected as well.

Suction versus moisture content values of TBJ units were obtained in the laboratory, using pressure plate, centrifuge and filter paper (Fredlund and Rahardjo 1993; Fredlund 1997; Rahardjo and Leong 1997; Bulut and Leong 2008; Murray and Sivakumar 2010; Fredlund et al. 2012) to build the Soil Water Retention Curve (SWRC). The Quickdraw Tensiometer was used in the field and in the laboratory as well. The practical reading range for the Quickdraw tensiometer starts from 0 kPa (saturated) to 77.5 and 74 kPa for elevations (above sea level) of 600 and 900 m, respectively (Communication of Soilmoisture 2012). Important factors to consider when using the tensiometer (Marinho et al. 2008; Tarantino et al. 2008) consist of having a good contact between the soil and the porous filter, avoiding the cavitation and interpreting when a good measurement is made.

**Fig. 8:**
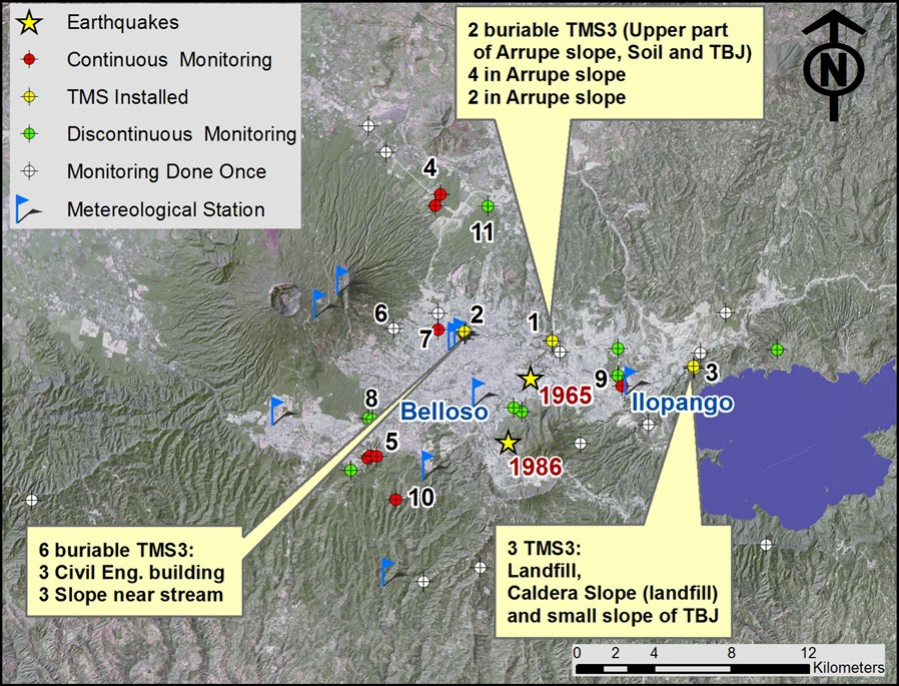
Location of points visited during research. Position of Belloso and Ilopango climatic stations is shown on the map. Location of 1986 and 1965 earthquakes are *yellow stars* from the earthquake catalogue of Salazar et al. (2013). Satellite image is a SPOT (2003) from the Ministerio de Medio Ambiente y Recursos Naturales

The Metropolitan Area of San Salvador (MASS) has a high population density (2848 hab/km^2^; Ministerio de Economia 2011) and an elevated criminal rate (Personal communication Observatorio Metropolitano 2012). For this reason, searching locations to regularly monitor and install the equipment in areas not classified as “unsafe” or visible to persons was a limitation. Only the TMS3 was deployed for continuous reading due to its small size and the capacity of its data logger (i.e. up to 500,000 values can be stored). In spite of the care and efforts, some of the installed equipment where damage or disturbed during the course of the project (points 2 and 3 of Fig. 8).

**Fig. 9:**
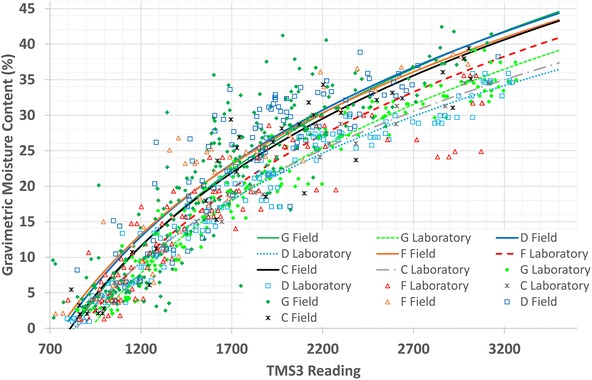
Field and laboratory calibration curve of TMS3 from all the monitoring places. Subdivision of the Tierra Blanca Joven pyroclastic units are identified by *letters G*, *F*, *D* and *C* (Fig. 3). The *points* are all the data gathered throughout the whole survey

During the first leg of the project (December 2012–December 2013) the first prototype TMS1 was used for intense monitoring. But the TMS1 suffered of insufficient mechanical resistance and climate conditions caused considerable degradation. For this reason around the end of July of 2013, the improved TMS3 sensor started to be used for monitoring. In spite of the different conditions of each monitored locations is possible to observe some tendency in the results (Fig. 9) for each unit for the field calibration (Chavez et al. 2014). Normally 20 min was the time required to attain equilibrium after installing the TMS sensor during the field calibration. The map of Fig. 8 shows some of the monitored places and devices installed in the area of study. Measurements using the TMS sensor and tensiometer were periodically made in some locations (Fig. 8) to observe changes of moisture and suction in the slope surfaces, where shallow flowslides are usual. A reconnaissance tour aided to locate typical slopes with interesting characteristics to build criteria of the behavior. The intention of this approach was to build a field calibration curve and test simple but reliable equipment that don’t need specialized training, in order to facilitate the habit of soil moisture monitoring (which is rarely done in some countries like El Salvador, due the lack of earth sciences professionals); and begin to build criteria about the pyroclastic behavior.

Climate information was collected from stations nearby, belonging to the network of the Ministry of Environmental and Natural Resources of El Salvador (http://www.snet.gob.sv/Geologia/pcbase2/parametros-mapa.php). Unfortunately, the UES station (Point 2 of Fig. 8) suffered a malfunction and the data was not available through most of the rainy season of 2013. For this reason, Ilopango and Belloso (Fig. 8) data was used for this research.

The TMS3 sensors are single measuring units that are physically independent. With TMS3 is possible to measure soil, vibrations and air temperature in addition to the soil moisture. The data are easily extracted with a device of the size of a small cell phone in a minute (Fig. 7). Also is possible to bury the equipment deep underground, as the external sensor can be in any distance/height of the central logger and remote data download is also possible (Personal communication of TOMST). The temperature is measured using a temperature sensor MAXIM/DALLAS Semiconductor DS7505U+, with resolution of 0.0625 °C and with accuracy of ±0.5 °C. TMS3 measures temperature at levels −10, 0 and +12 cm relative to soil surface when installed. TMS3 can record the data in different intervals (15, 10, 5 and 1 min). For this project, the mark of 10 min was chosen, as is the same interval used by most of the climatic stations and to secure the batteries life.

Besides the field calibration, a laboratory calibration using disturbed TBJ samples was generated. For each field visit or moisture content in the laboratory, a measurement with TMS3 was taken. Other possibility to make the calibration curve in the laboratory is to use a special apparatus (Personal communication of Šanda 2014), based on principles of indirect gravimetry. Saturated consolidated disturbed samples are weighted during evaporation (using Tedea Huntleigh loading cells). The drying process is enhanced by forced air ventilation throught the vertical profile of the sample. If both methodologies are compared, similar results are noted for TBJ results and a calibration set for typical soils created by Jankovec et al. (2013).

After plotting the field and lab points it was necessary the use of statistics to obtain the calibration curves. In the case of field calibration results, it was important to understand the influence of temperature T1, T2 and T3 (at levels −10, 0 and +12 cm relative to soil surface respectively). The software Statgraphics Sigma Express was used for processing the TMS3 data. To explain the field observations the analysis of variance was used. Also to obtain the calibration curves, simple and multiple regressions curve fitting were obtained as well. After comparing the fitting with other several curvilinear models, the curve fitting model with the highest R-Squared (R^2^) value was chosen.

Comparison between TMS3 lab and field calibration curves using (results from a combination of all monitoring locations for each TBJ unit) are shown in Fig. 9 and Table 1 where the curve equations are presented. Similarities between the field calibration curves of the monitored TBJ units are evident. The information is composed by data from the years (2013–2014) that include the cases when the slope was drying or saturating. For the C unit only one slope was monitored (point 4 of Fig. 8).

**Table 1:** Equations obtained from field and lab calibration of different TBJ units including the R-squared statistic

TBJ unit	G	R^2^
Field	−207.73 + 30.9205ln(TMS3)	95.36
Laboratory	−199.869 + 29.2857ln(TMS3)	98.21

TBJ unit	F	R^2^
Field	−187.084 + 28.249ln(TMS3)	92.05
Laboratory	−199.047 + 29.4028ln(TMS3)	96.21

TBJ unit	D	R^2^
Field	−195.35 + 29.3735ln(TMS3)	82.43
Laboratory	−170.794 + 25.3951ln(TMS3)	97.74

TBJ unit	C	R^2^
Field	−198.437 + 29.6204ln(TMS3)	95.71
Laboratory	−177.016 + 26.2691ln(TMS3)	99.39

Curves obtained in field and lab show a difference in the gravimetric moisture content (up to 7 %) that could be associated to hysteresis (drying or wetting process). In the case of the laboratory calibration is not possible to simulate or replicate the constant change of moisture content that goes constantly in the slopes and to replicate exactly the stress states. Also it was observed that during installation of the TMS3 there is a chance of having poor contact with the soil during measurement. When the pyroclastic is saturated, liquefaction could happen during installation and change the results. In the Fig. 9 is obvious that bigger differences are more evident when the soil is close to saturation. Currently is not clear the influence of osmotic suction in the pyroclastic behavior. This issue has to be study in more detail.

The reading surface of TMS3 has a longitude of 12 cm therefore, the obtained moisture is an average value. To check the differences in moisture as the deep changes, different values of moisture for a depth of 6 cm, and 12 cm were taken in different monitored locations. A difference of 4.57 % of moisture (mean value) was obtained with a standard deviation of 2.98.

## Results

**Fig. 10:**
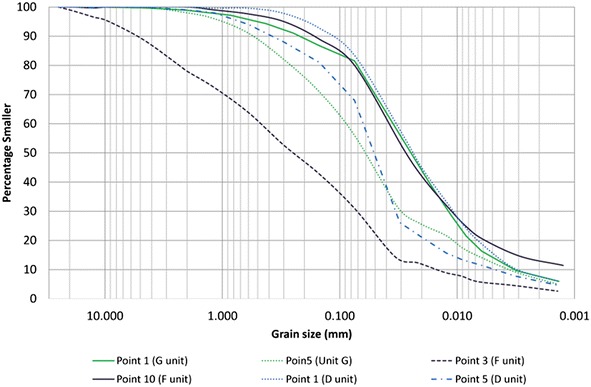
Grain size curves for all the monitored units of TBJ (showing the coarser and finer boundaries only). Divisions of units according to (Fig. 3)

Grain size curves intervals (coarser and finer) of the monitored units are presented in Fig. 10. For G and D units of TBJ the point 5 (Fig. 8) were coarser than the Arrupe location (point 1 of Fig. 8). This can be associated to the thickness of the density current flows that are thicker in the latter point (Fig. 2) and possible development of co-ignimbrite plume from the density current flows. In the case of the F unit is coarser close to the Ilopango caldera (point 3 of Fig. 8) that in the point 10. The pyroclastics are classified as a sandy silt or silty sand, based on the Unified Soil Classification System (USCS), being very heterogeneous and changing its properties according to location, facie and moisture content (Bommer et al. 2002).

The gravimetric moisture content of TBJ during the time frame of this research vary between 2 and 42 %. The specific gravity is among 2.25–2.5 and the void ratios 0.6–1.6, corresponding to average porosities of 37.5–61.53 % (Rolo et al. 2004; Hernandez 2004). The bulk density vary between 0.85 and 1.17 g/cm^3^.

**Fig. 11:**
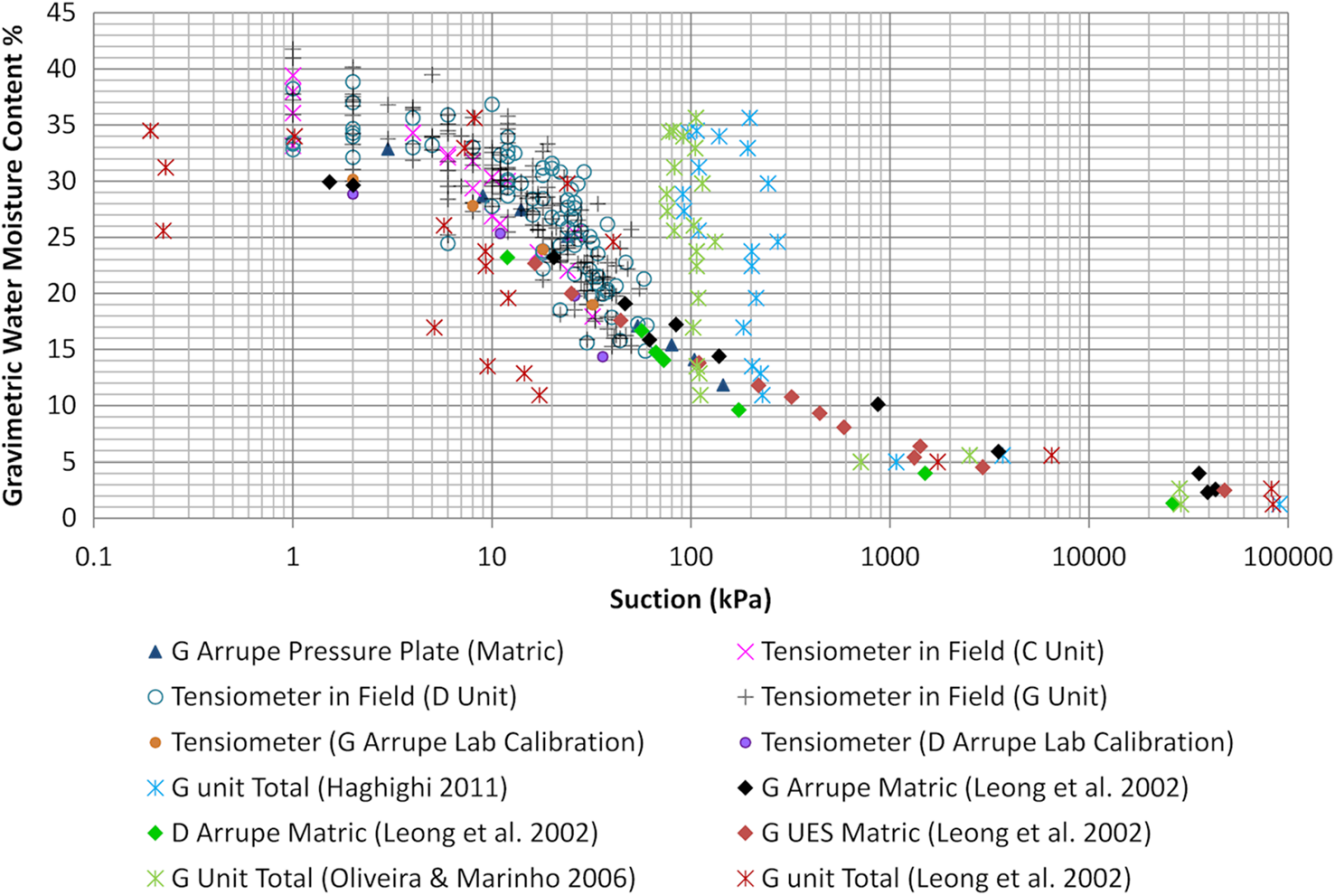
Matric and total suction measurements for TBJ Modified from Chavez et al. (2013)

The TBJ units chosen for monitoring were mainly G, F and D units due to its importance as they are the shallowest deposits. Chavez et al. (2013) completed the Soil Water Retention Curve (SWRC) for these TBJ units. Measurements in the laboratory of matric and total suction vary between 0 and >20,000 kPa (Fig. 11). The equations used for the paper filter method (which proven to be the more practical method) are presented in Table 2. Comparison between the matric suction obtained in laboratory and the field indicate hysteresis in the field, as drying or saturation processes were happening during field monitoring according to its moisture content.

**Table 2:** Filter paper equations of different authors

References	Suction	w (%) range	Log10 (suction) (kPa)
ASTM D5298	Total and matric	w < 45.3	5.327 − 0.0779w
ASTM D5298	Total and matric	w > 45.3	2.412 − 0.0135w
Chandler et al. (1992)	Matric	w < 47	4.842 − 0.0622w
Chandler et al. (1992)	Matric	w > 47	6.050 − 2.48 Log w
Oliveira and Marinho (2006)	Matric and total	w < 33	4.83 − 0.0839w
Oliveira and Marinho (2006)	Matric and total	w > 33	2.57 − 0.0154w
Leong et al. (2002)	Matric	w < 47	4.945 − 0.0673w
		w > 47	2.909 − 0.0229w
Leong et al. (2002)	Total	w < 26	5.31 − 0.0879w
		w > 26	8.778 − 0.222w

Reference	Suction	Suction range	ln (suction) (kPa)
Haghighi (2011)	Total and matric	ψ < 500 kPa	33.97w^−0.33^ − 4.55T^0.04^
		ψ > 500 kPa	−1.23(w^0.56^ + T^0.19^) + 16.48

w = Gravimetric water content; ψ = suction (kPa); T = temperature (°C)

**Fig. 12:**
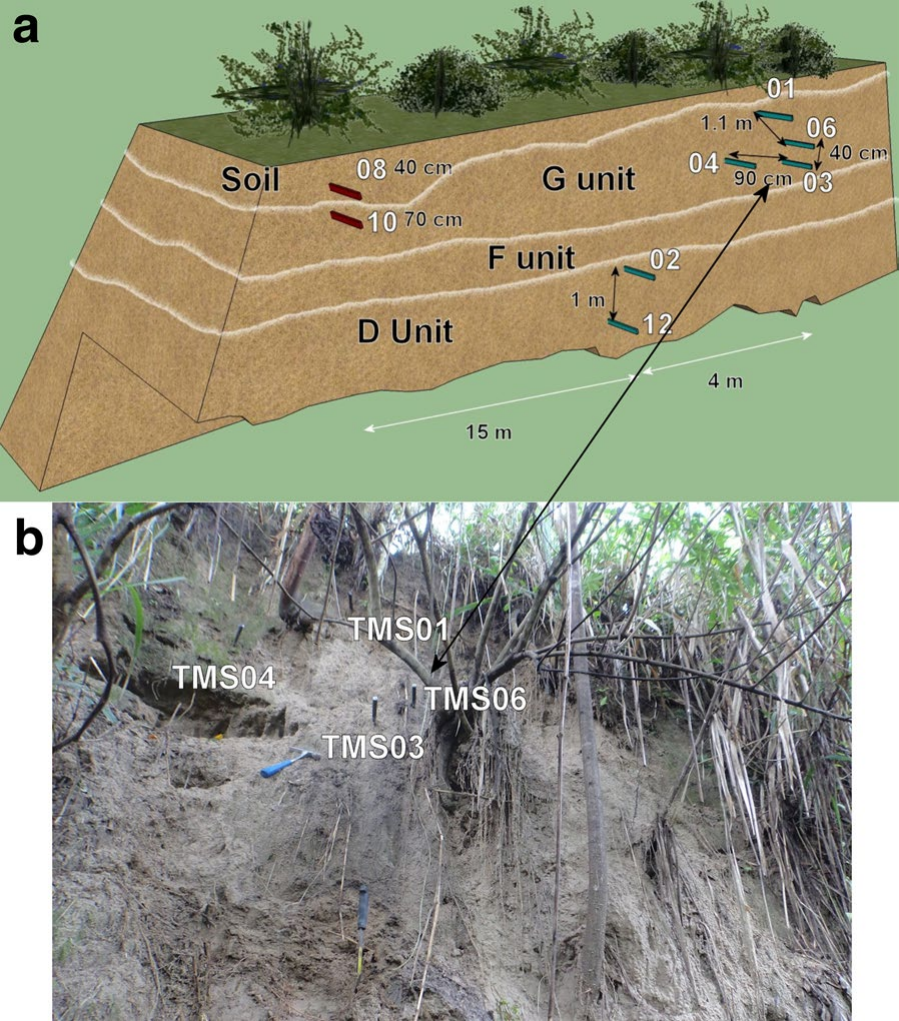
Scheme with TMS3 installed in the Arrupe location (average slope inclination is 76.4°). **a** *Color red* indicates devices that are buried and *blue green* are installed in the surface. **b** The TMS3 installed after one flowslide in June 18, 2014

**Fig. 13:**
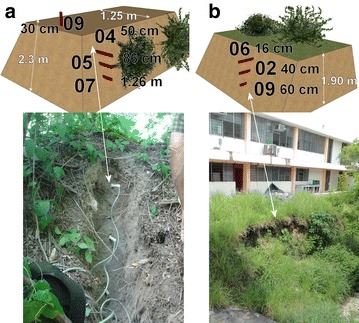
Scheme with TMS3 installed in the UES location. **a** Unit F, is point 2 (average inclination is 60° in the area of the installed TMS3 and the depth to the river is 20 m) and **b** unit G (average inclination is 87.5°). *Color red* indicates devices that are buried

In El Salvador, the rain season goes from May to October. Historically the months of June, September and October have the maximum precipitation. Three to six days of constant rain known as “temporales” could be problematic in these months. Usually periods without rain between July and August occur that could last ten to fifteen continuous days (Garcia 2009). Of the monitored locations of the AMSS (Fig. 8), point 1 (Arrupe) and 2 (UES) were chosen for continuous monitoring with TMS3 (buriable and surface). Close to the Ilopango caldera equipment was installed for a short time (point 3 of Fig. 8). A scheme of the main points is presented in Figs. 12 and 13 with TBJ units and installed equipment.

The average annual precipitation of Ilopango and Belloso meteorological stations is 1800 mm (Fig. 8). The average annual potential evaporation is around 1750–1850 mm. The average annual real evaporation is around 800–900 mm. The average air temperature is 23 C° (Ministerio de Medio Ambiente y Recursos Naturales 2006). Groundwater level of Arrupe is at 65 and 112 m for UES (Personal comunication of Arevalo et al. 2007). Most of the slopes have some kind of anthropogenic intervention (road cut, landfill, close to urban project) and belong to erosion hillsides and Badlands according to Chavez et al. 2014. For this reason, the susceptibility to denudation is higher in the Arrupe location (Badlands and road cut), as was observed during the monitoring time.

**Fig. 14:**
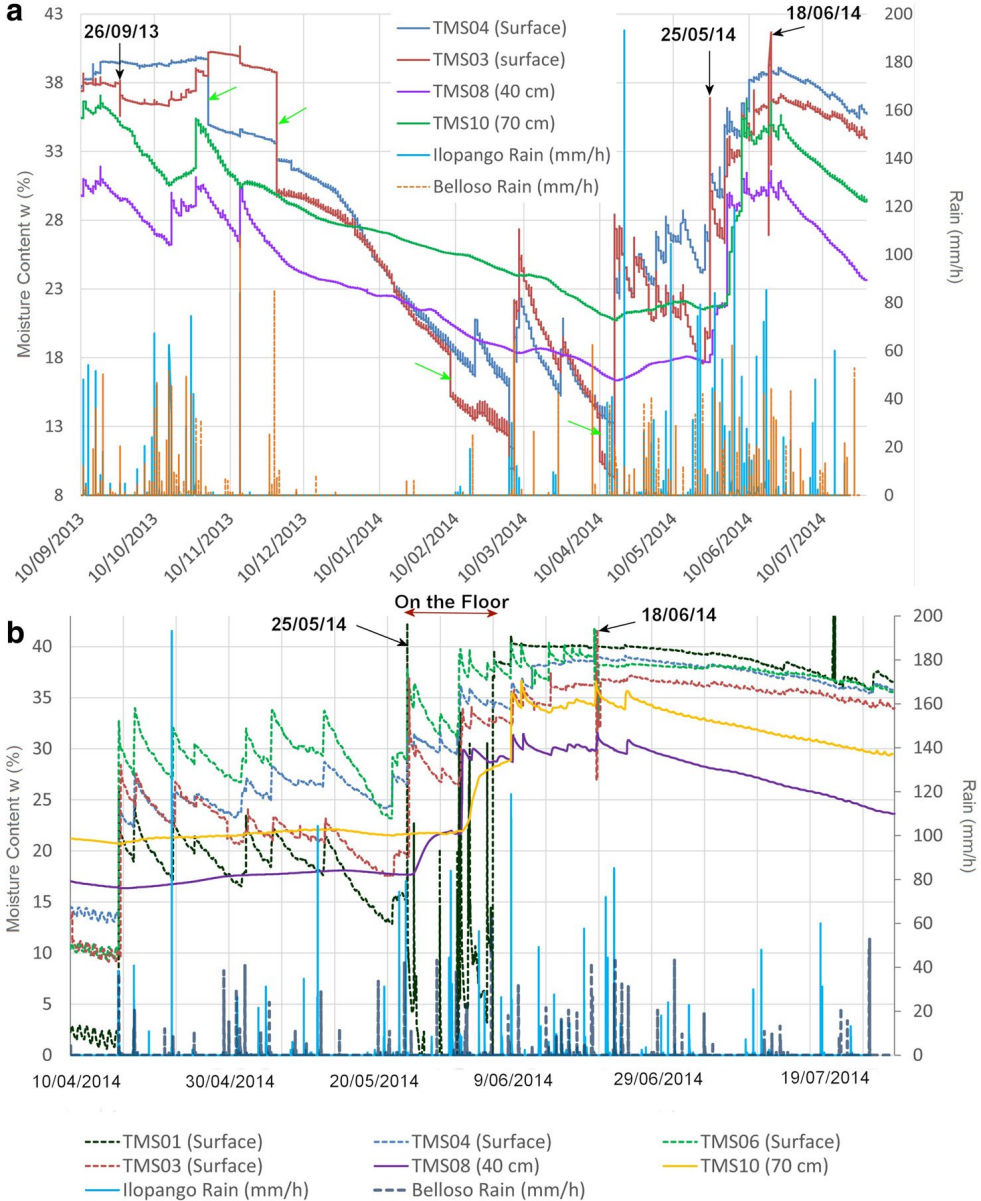
TMS3 results of the installed in the Arrupe location according to installation date. In **a** only four devices were installed and in **b** is presented the results of six installed devices. Dates indicate flowslides. With *green arrows* are illustrated the lack of contact during extraction of information from the device. TMS04 was installed in an area with presence of biological soil crusts (BSCs). TMS01 was on the floor after 25/05/14 rain

**Fig. 15:**
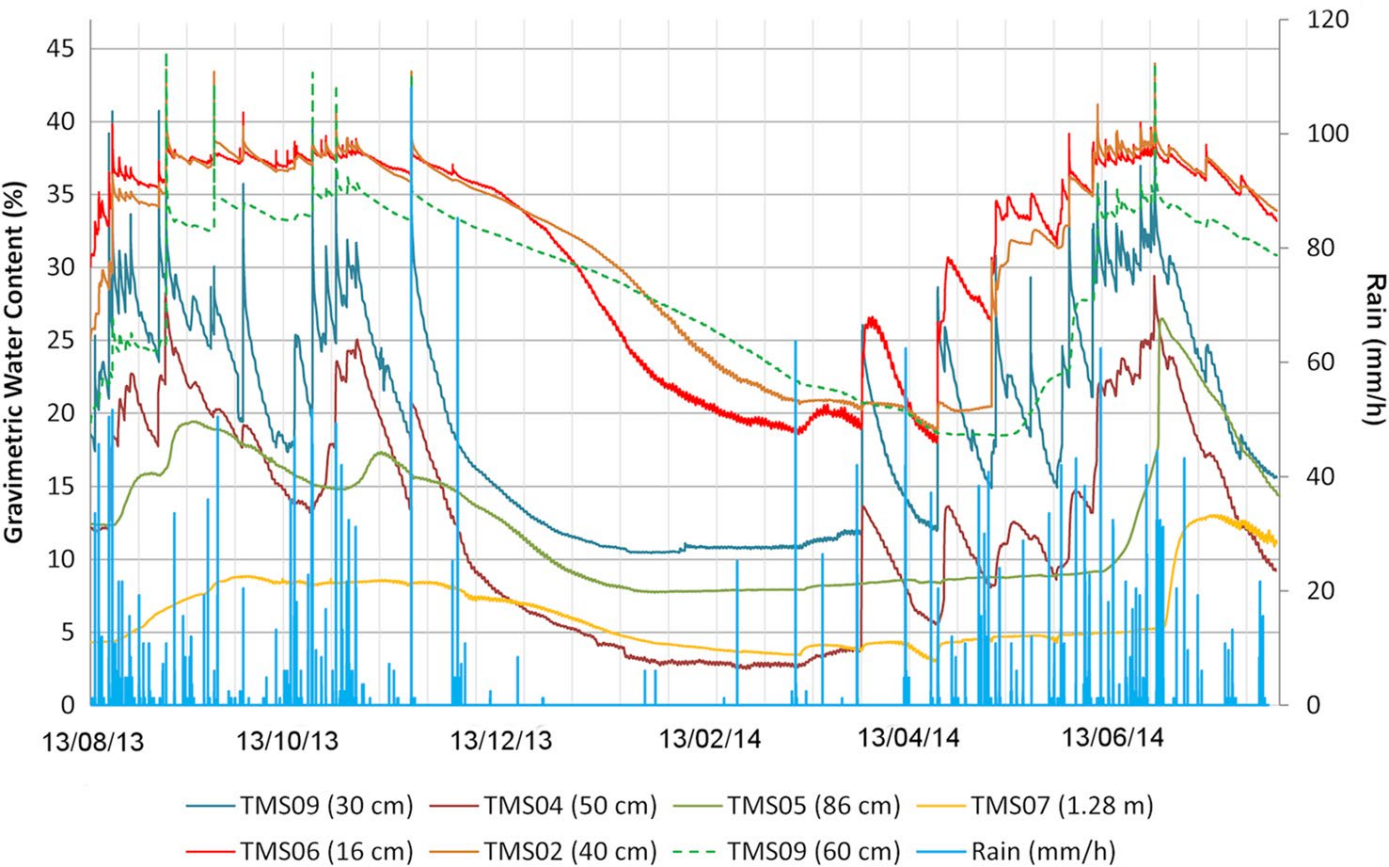
Results of the installed TMS in the UES location. TMS number 07, 05, 04 and 09 were installed in a dome-watershed like surface that prevented infiltration

**Fig. 16:**
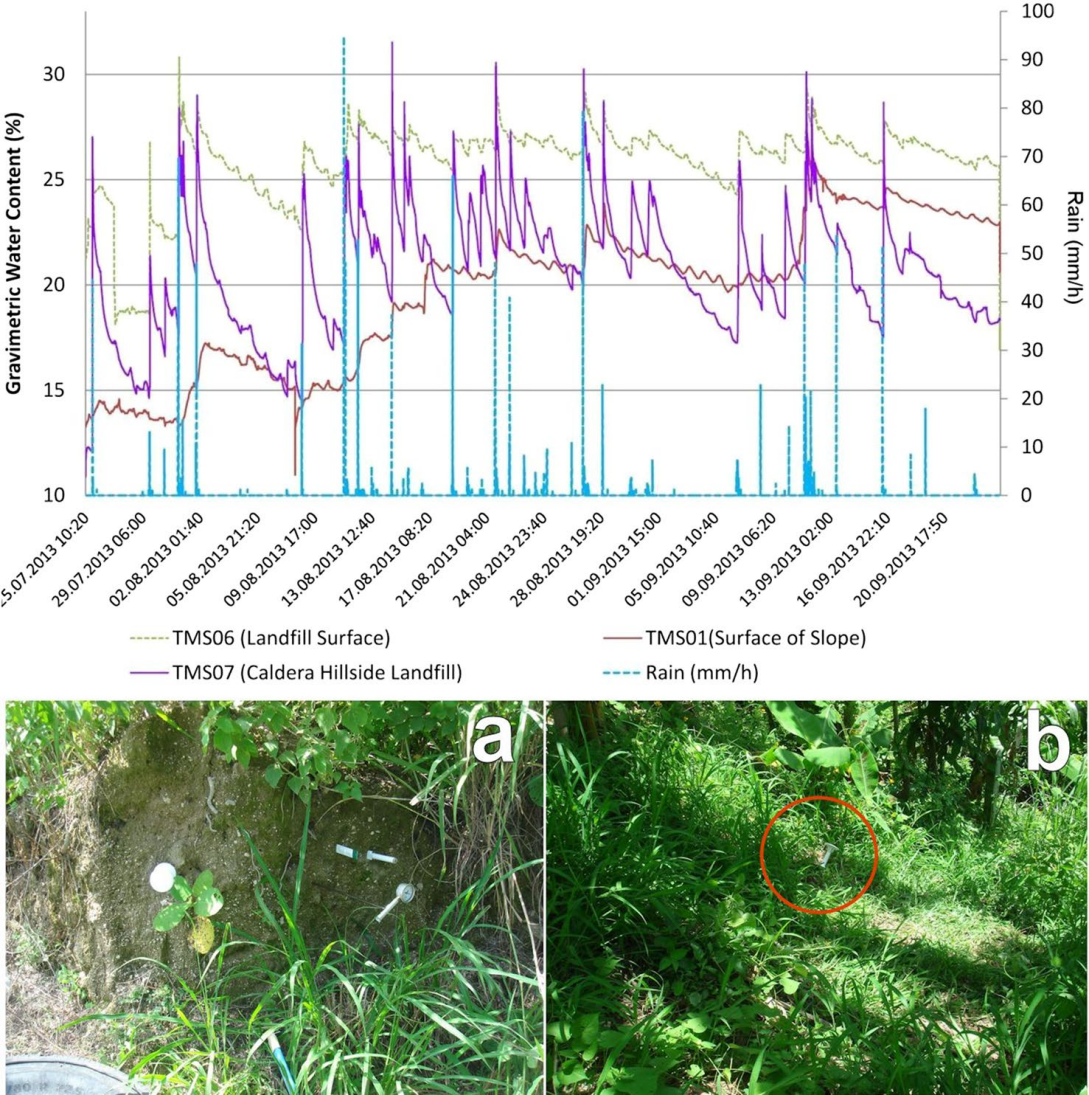
Scheme with TMS3 installed in the Ilopango Caldera location (point 3 of Fig. 8). **a** TMS01 installed in F unit on Ilopango caldera hillslope, **b** TMS07 installed in landfill on a Ilopango caldera hillslope. TMS06 was installed on a landfill (20 m south of TMS01)

In Figs. 14, 15 and 16 are presented the moisture content results of the installed TMS3 along with information of the precipitation during the project period (calibration curves are presented in Fig. 9 being the field results used).

During the monitoring it was observed in all the slopes an important presence of biological soil crusts (BSCs) (Xiao et al. 2011) a complex mosaic of soil, green algae, lichens, mosses, micro-fungi, cyanobacteria and other bacteria covering the slopes surface. The crust could be also related to the impact of the raindrops (Cheng et al. 2008). It was observed that this crust can protect somehow the steep slopes reducing the runoff process and mass wasting processes. Also the growth of BSCs (e.g. moss and lichen) helped to retain the moisture content of the slopes (Figs. 6, 14) this was observed in the field and recorded by the TMS3.

The pyroclastics tend to keep the humidity inside the slope despite that the surface crust could be dry. In some areas, the D unit of TBJ (more fine grain size) remained with humidity most of the year (BSCs are active through all year) and generally in these areas, traces of weathering (oxides) are found. Smectite are found in the minor clay fraction of TBJ samples (Chavez et al. 2014).

Comparisons between close installed TMS03 and TMS04 (90 cm away) in the Arrupe location (Point 1 of Fig. 8; Figs. 12, 14) proven the importance between an uncovered area compared to one protected by BSCs like TMS04. But also temperatures differences linked to spots of shadow due trees and direct sun exposition can be the responsible of moisture (suction) differences through the entire slope. Soil use, topography (Fan and Hsiao 2012), evapotranspiration, cracks, temperature, winds, moisture and vegetation are influencing factors in moisture content as well. Usually around July and August there are some days without rains that could reduce the landslide susceptibility. According to TMS results and regular field inspections, it appears that the runoff connected with the slope topography is important in the saturation of different areas. The slope goes from states close to saturation to dry regularly.

Different behavior is observed in the buried TMS08 (40 cm in organic soil) and TMS10 (70 cm in TBJ) as an opposite behavior was noted, as the surface layer (organic soil) was drier than TBJ (layer below) through all the monitoring time.

The F unit (pyroclastic flows) of TBJ (Fig. 3) in different areas of the MASS contains large pieces of rocks and pumice (it can be up to 52 cm in some areas, according to Hernandez 2004). This continuously prevented a good contact for the tensiometer porous ceramic sensing tip and the TMS sensor. For this motive, the results were particularly poor in this TBJ unit and it was not possible to build a good calibration curve for the TMS3 or suction measurement in most of the proximal facie sites (Fig. 9). In a dry state the pyroclastics are stiff but soften when its state is close to saturation. A core tool was needed for introducing the tensiometer and TMS3 sensor; sometimes this disturbed the soil and caused lack of contact when the equipment was introduced for measuring.

**Fig. 17:**
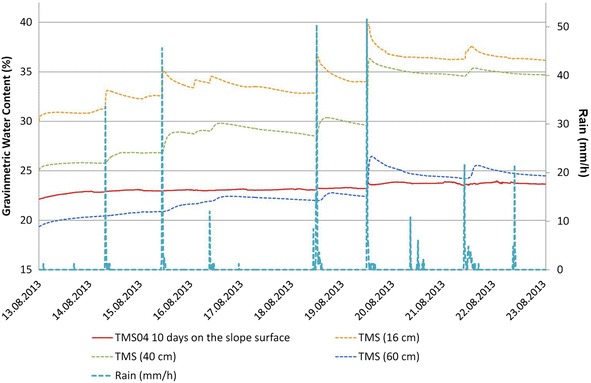
Differences between TMS installed in the face of a slope (TMS04) and others buried in the slope (UES location)

In the UES location (point 2 in Fig. 8; Figs. 13b, 15, a comparison was made for 10 days between the TMS3 buried underground and one installed on the face of the slope (Fig. 17). Since the area of installation was disturbed, the TMS on the surface was retired after the 10th day. Differences are visible in the moisture content as the rain might not impact directly on the face of the slope (depending on direction of winds and displacement of rainstorm). In the case of the buried TMS, the rainwater was accumulated in the surface and infiltration was allowed, but the process to reach the slope face is slow as the permeability depends of the moisture content.

**Fig. 18:**
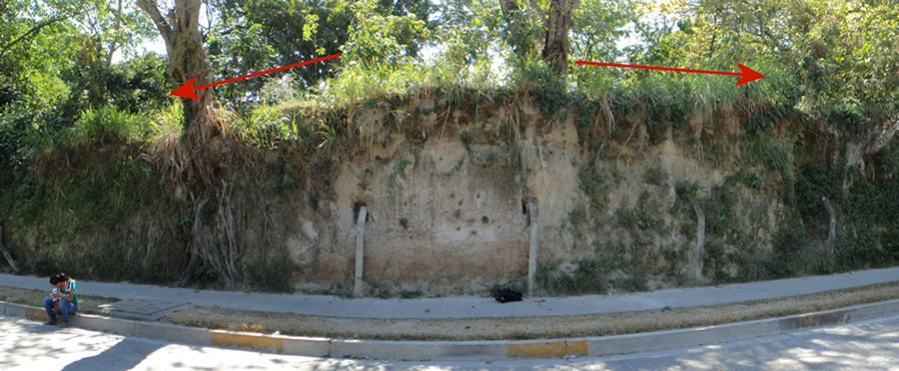
Topography in the surface that avoid saturation of slopes. Location is point 5 in Fig. 8

The surface topography of the upper part of the slopes influence the rainwater infiltration, for example slopes with a dome-watershed like surface (Figs. 13a, 15, 18), prevent water infiltration and are always drier than adjacent areas. This situation is more evident (Figs. 13a, 15) with TMS07 (1.20 m) that didn’t show important changes during the monitoring time. Most of the rainwater becomes runoff. The water only infiltrates the surface (as observed in Figs. 14, 15) and then is taken out by groundwater flow or evapotranspiration.

The TMS buried in a slope (Figs. 13b, 15) with an uneven surface (where rainwater can infiltrate show less significant differences between the surface (TMS06) and more deeper device (TMS09) which store a constant moisture during the research (20 % approx.), being the TMS06 (closer to the surface) with more significant changes.

**Fig. 19:**
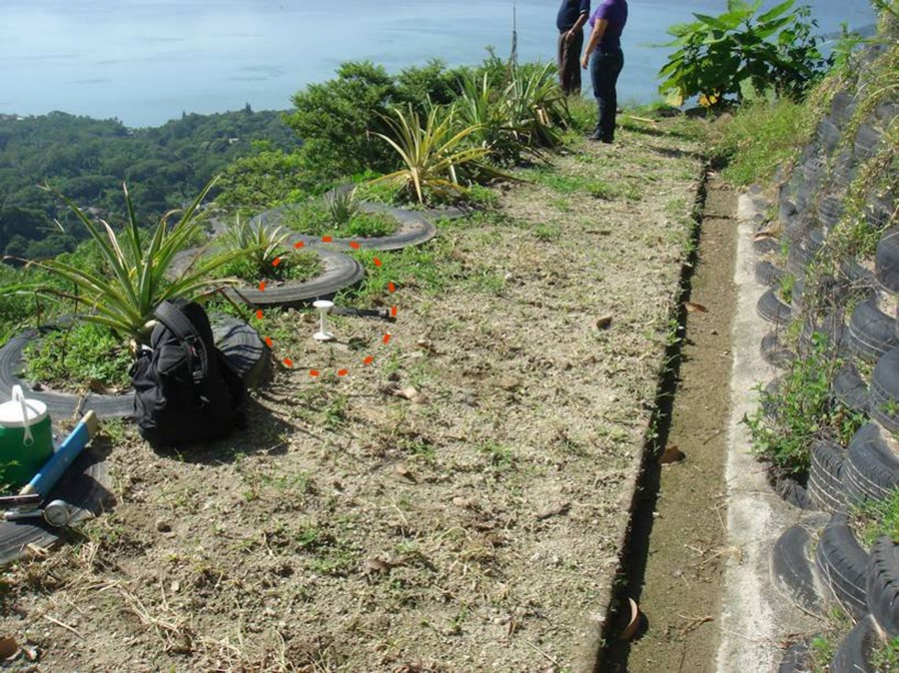
TMS06 (Fig. 17) installed on the surface of a landfill. A typical slope mitigation work in the country, using old car tires. Location is in the hillside of Ilopango Caldera (I the background is visible the lake filling the caldera)

In the Ilopango caldera the TMS06 was installed on the surface of a landfill (a typical design in the country, using old car tires) (point 3 of Fig. 8; Figs. 16, 19) without vegetation. Close to this location (20 m south) TMS01 was on a slope of F unit TBJ. On the North (approximately 100 m) TMS07 was installed on a landfill covering a steep caldera slope. It draws attention that most of the time the landfill (where the TMS06 was installed), shows constant moisture content, it must be related to the lack of coverage of vegetation and landfill drainage. TMS01, as water infiltrates shows a constant increase in moisture content. TMS07 results once again indicate the importance of slope inclination. Vegetation and topography have a role in the behavior and stability of the slopes as the rainwater is not able to accumulate for a long time and the saturation-drying process is continuous. Next to this point a debris flow occur as the runoff water concentrated in one sector of the slope during 2009 Ida storm.

During the monitored time several events of small shallow sand/silt/flowslides happened in the instrumented slope of Arrupe (six events of them affected the area of installed devices) (Fig. 14). For this reason more TMS (TMS04, TMS06 and TMS01) were installed on different dates to gather more information (at the start there was only one installed, TMS03). The date and hour of occurrence of the flowslides in the site was derived from the measurement of temperature (abrupt changes), moisture measurement of the TMS, vibrations, field observation and the precipitation characteristics during the evaluated event. The slope has an inclination of 70°–90°. The approximate volume of the flowslides was between 0.0016 and 0.6 m^3^. It was observed that usually the flowslides were initiated by a particular rain (the smaller ones connected with >10 mm/h rain and bigger size flowslides with a 40 mm/h rain for Belloso station and >58 mm/h for Ilopango station). Gravimetric moisture content >30 %. Suction measures before and after the flowslides were <10 kPa.

**Fig. 20:**
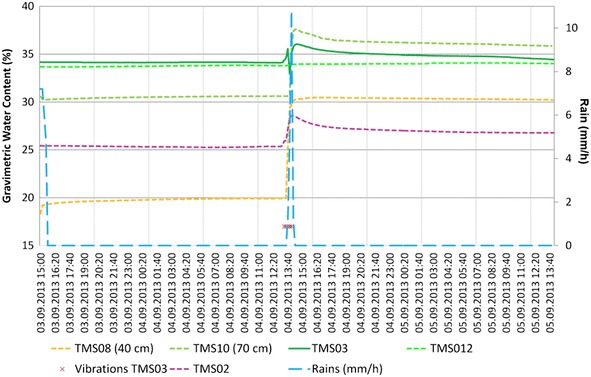
Small flowslide (September 4 of 2013) in Arrupe slope that partially affected TMS03

**Fig. 21:**
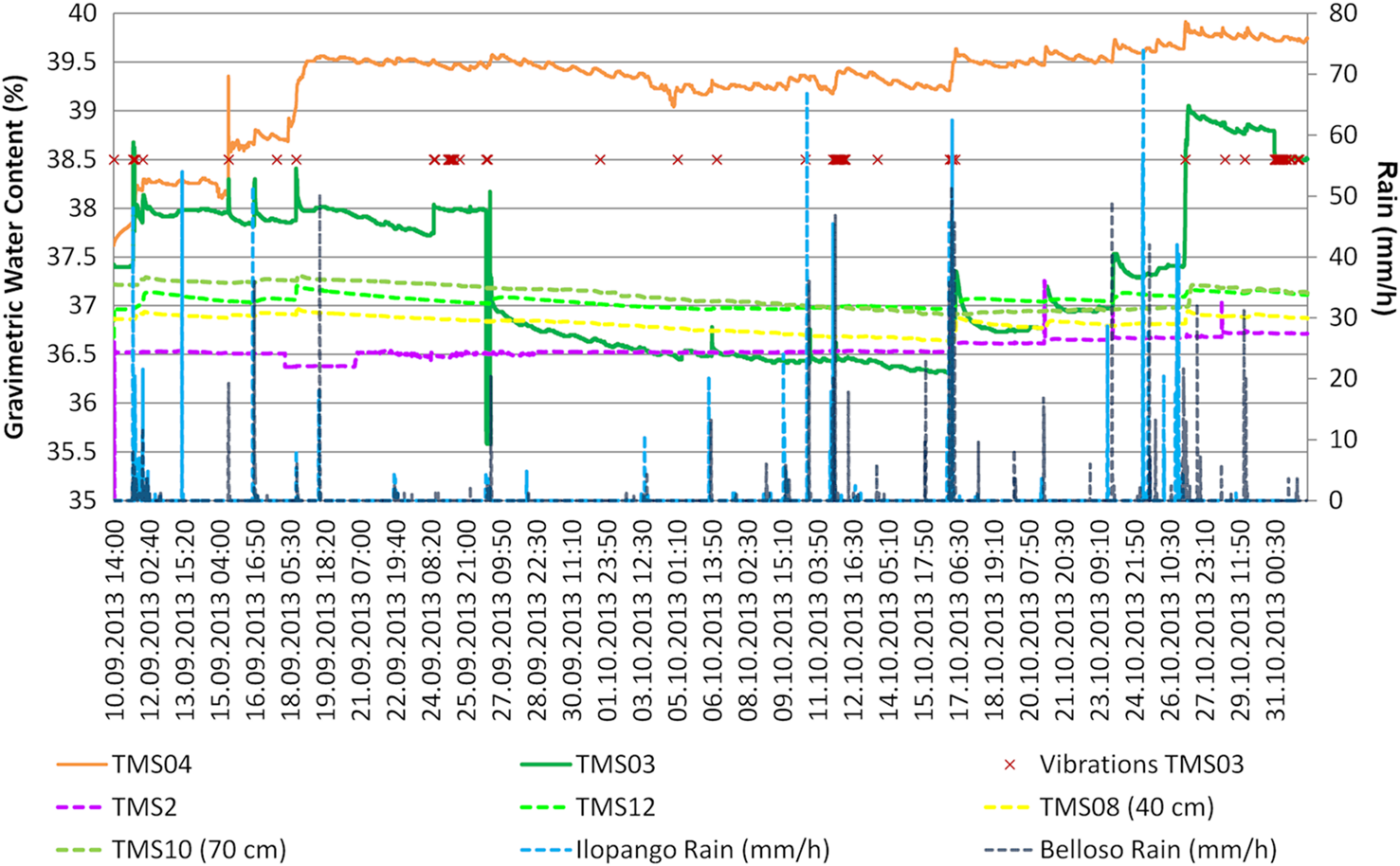
Small flowslide (September 26 of 2013) in Arrupe slope that partially affected TMS03. Notice that TMS04 located in a moss covered surface keeps moisture content higher and constant through the time

**Fig. 22:**
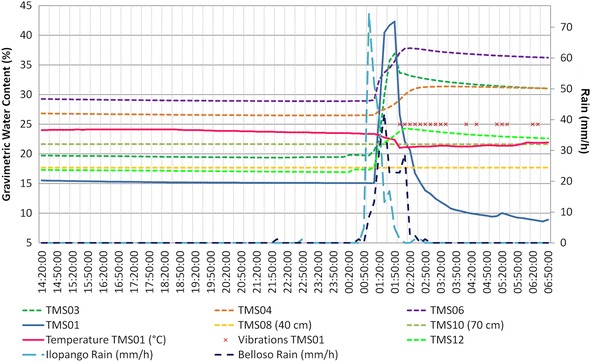
Small flowslide (May 25 of 2014) in Arrupe slope that affected TMS01. The device fell on the ground as a consequence

**Fig. 23:**
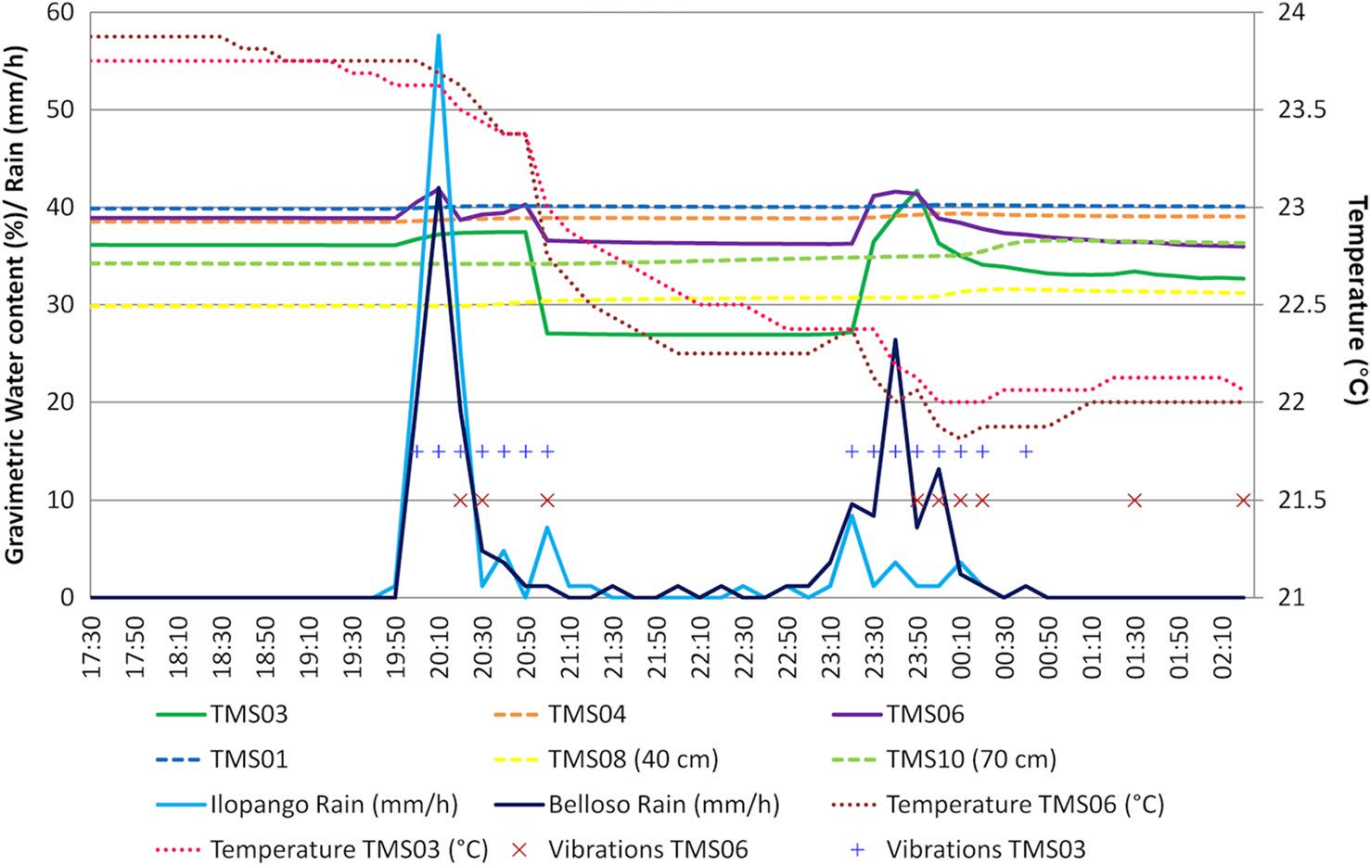
Small flowslide (June 18 of 2014) in Arrupe slope that affected TMS06 and TMS03. The devices fell on the ground as a consequence

The events of 4/09/13 (Fig. 20); 26/09/13 (Fig. 21) (smaller) were associated to flowslides deposits that ran into the TMS (the water content decrease and then increase again). In the case of the flowslides of 25/05/24 (Fig. 22) and 18/06/14 (Fig. 23) the affected TMS fallen into the ground with the deposits of the flowslides.

The failure area occur along the root area, fissure or crack areas and where water runoff concentrates (connected to topography of the hill or slope). Other aspect that can influence the flowslides is the seepage. In TBJ it was observed that there are very small soil pipes (related to animals, roots, internal erosion, etc.) which could act as preferential paths of water infiltration and saturation of the matrix (Montrasio and Valentino 2007). The slope was affected in different dates by flow slides through its entire surface. It appears that it’s an ongoing process that changes the morphology of TBJ deposits constantly.

More sand/silt/flowslides occurred in the other monitored places the same days as in the Arrupe location, showing the same similarities mentioned before. Values of H/L between 2.8 and 0.8 were common for small flows in the monitored sites of Tierra Blanca Joven pyroclastics during this research. Most of the sand/silt flowslides in TBJ are shallow with low volume. But also a debris flowslide/debris avalanche can take place (Fig. 4).

## Discussion

The urban areas resting on top of unsaturated pyroclastic soils (e.g. TBJ) or close to the streams are prone to mass wasting processes and collapse. The problems experimented in the Metropolitan Area of San Salvador are the failure of infrastructure like water pipelines, housing developments and roads), also sand/silt/debris flowslide and debris avalanche and erosion (sheet run off, vertical, lateral, planar, rill, gully and underground erosion). The intention of the present research is to understand the behavior and characteristics of a problematic pyroclastic soil (TBJ) for future application in the land use and have a better planning for the mitigation. Field monitoring prove to be important to recognize the external factors that affect the saturation of the pyroclastic soil.

Comparison (Fig. 11) between the lab and field results of different methods (filter paper, pressure plate, tensiometer and centrifuge) revealed a good correspondence. The consolidated state and the grain size distribution of TBJ might influence the observed differences in the SWRC results. For the filter paper, different equations (Bicalho et al. 2007) were used (e.g. ASTM D5298; Chandler et al. 1992; Leong et al. 2002 and Oliveira and Marinho 2006; Table 2), but if compared with the pressure plate results and field tensiometer results, the Leong et al. (2002) equations show a better fit for matric suction; the results of other equations begin to detach after 100 kPa. The results of the mathematical models (an equation for the whole range of suction; e.g. Van Genuchten 1980; Brooks and Corey 1964; Kosugi 1996; Durner 1994; Seki 2007) used to describe the SWRC of TBJ, indicate that the filter paper data also has a better coefficient of determination (Van Genuchten had the best R^2^) than the values obtained with the pressure plate. The filter paper technique proves to be the most practical. Is able to measure matric and total suction, time needed is shorter and is possible to measure suction for almost the whole range. But for suction values <10 kPa (Bicalho et al. 2007) the pressure plate results are required.

There have been attempts In El Salvador to have a landslide threshold (debris flows) linked with the rain quantity, but there was not continuity in the collection of data and a comparison with smaller scale sand/silt/debris flowslides, which are more usual and occur with a greater density in the territory during the rainy season. For the Ministry of Environmental and Natural Resources of El Salvador (Ministerio de Medio Ambiente y Recursos Naturales 2005) the threshold for initiating a debris flow are 24 h accumulated rain >100 mm and 15 days accumulated rain >240 mm. With this research, a study of flowslide threshold for pyroclastic has initiated. More research is needed it to improve the results and build some early warning procedure according to its characteristics.

Is crucial the information of the antecedent precipitations, since a 24 h rainfall event is not enough to trigger landslides (Pagano et al. 2010). For example, during Hurricane Ida (2009) several sand/silt/debris flowslide/debris avalanche were initiated in the scarps of Ilopango Caldera (covered by TBJ, Figs. 4, 5), but for the event DT12E (2011) there were not landslides observed, in spite having more accumulated rain (24 h) than Hurricane Ida. These recent landslides that have taken place mostly close to Ilopango Caldera, might be connected with a large volume of material in the streams ready to be mobilized or to deposits in the bed stream that is impacted by smaller landslide mass (drained or undrained loading) producing fluidization (Hungr et al. 2013). In addition, a time of preparation for anthropic, erosion mechanical and chemical weathering processes might be needed to set the next failure area and accumulation of deposits inside the drainage systems.

Just the information of rainfall (thresholds by other authors are summarized in the background section) is not enough to predict or locate the affected areas and how they evolve (e.g. hyperconcentrated, flowslides or debris flows) (Cascini et al. 2013). For example is usual that neighboring areas with similar characteristics behave different during a rain or earthquake that initiates one or two important mass movements.

Aside from climatic conditions (rainfall, wind and temperature variations affecting evapotranspiration and soil moisture); in the case of the monitored points (with installed TMS3) in the MASS, the observed flowslides were connected with local conditions like hillslope aspect, morphology, geology, hydrology, seismicity, weathering, grain size, vegetation, cracks, mineral, anthropic factors and soil crust and BSCs (Wieczorek and Thomas 2010; Cascini et al. 2013). As slopes are with low suction during long periods the antecedent rainfalls are important (Pagano et al. 2010). Topography is important since it can help to accumulate water into the soil in some areas, for example where the water runoff concentrates and in areas with topographical depressions (Kim 2011).

**Fig. 24:**
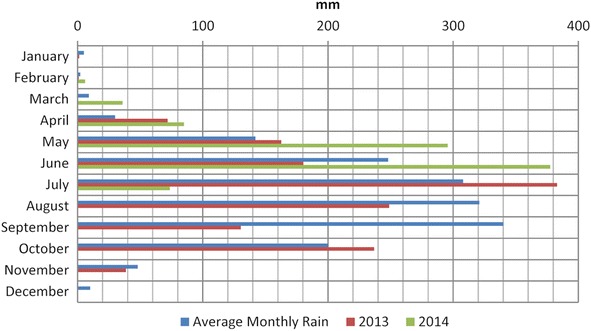
Average monthly precipitation for Ilopango Station. Including the monitoring data of 2013–2014 (Ministerio de Medio Ambiente y Recursos Naturales 2006)

**Fig. 25:**
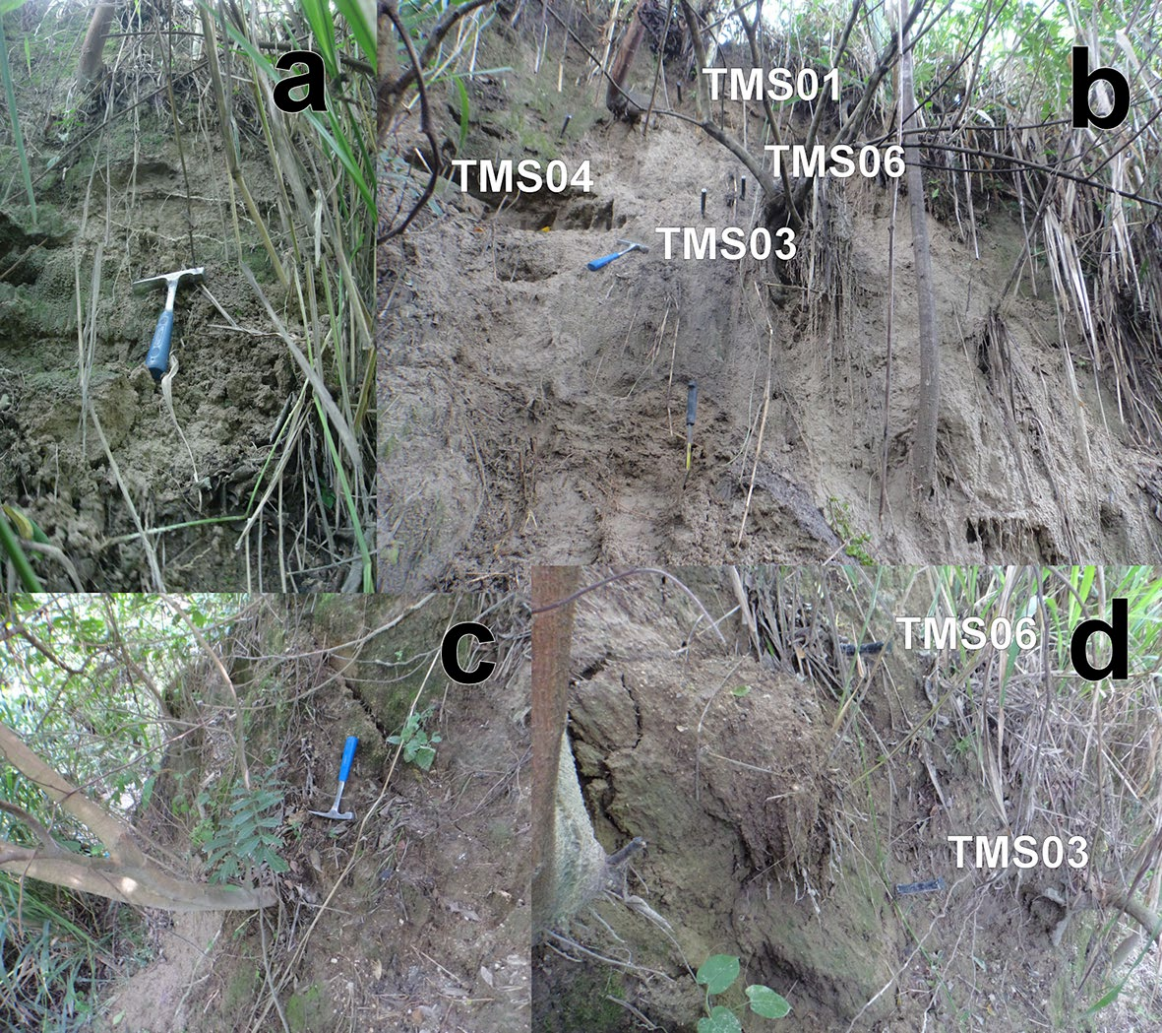
Above is presented the flowslides of 4/09/13 (**a**) and 18/06/14 (**b**). **c**, **d** Cracks in the slope before 18/06/14 flowslide

**Fig. 26:**
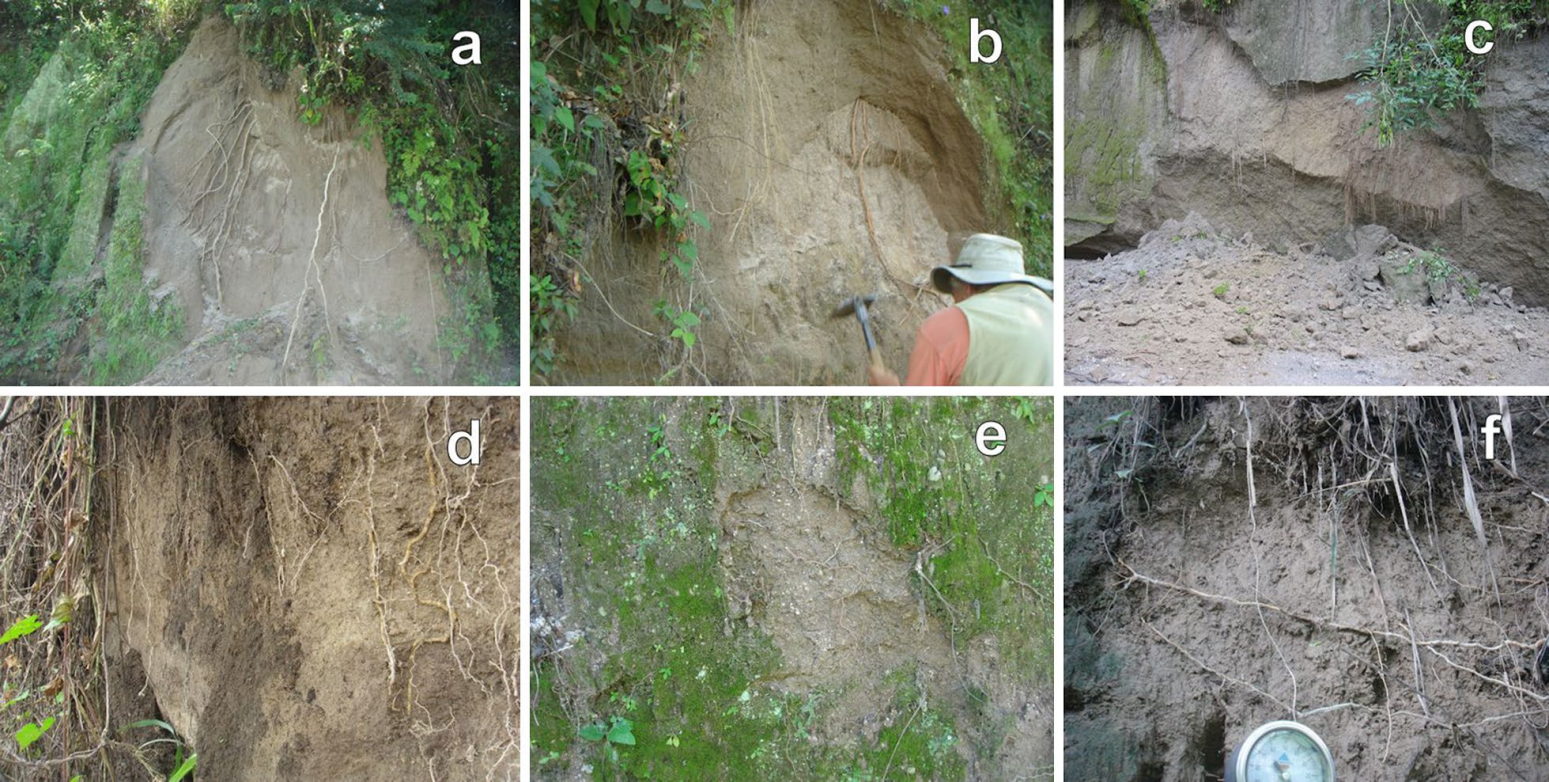
Flowslides and failure of undisturbed block of material in the root area in TBJ slopes. From Fig. 8 photo **a**, **b** located south of point 5; **c** north of Ilopango meteorological station; **d** point 5; **e** point 8 and **f** point 1

The months that are more prone to be affected by liquefaction and flowslides are June–September but it can vary according to climatic and anthropogenic conditions each year (Fig. 24). Similarly as the case of studies in Brazil (Savage and Baum 2010) the failure of TBJ slopes are shallow occurring bellow or parallel to the thin root mat (Figs. 25, 26) and the suction decreased (saturation) more in the surface, after an intense rainfall. Usually changes in suction are not severe at deeper levels (Figs. 14, 15). In 2014, intense rains happened between May and June and flowslides took place in the slopes (Fig. 24). During 2015 a drought delayed the start of the rainy season and for this reason the saturation was not reached until the end of the rainy season.

For the monitored flowslides in TBJ pyroclastics (Table 3) the threshold for initiating significant flowslides was >20 mm for the whole event, 15 days of accumulated rain >87 mm and 30 days of accumulated rain >148 mm. More research is needed to have a representative data including other factors that can trigger the flowslides. The slope behavior was monitored during the dry and wet seasons allowing that changes could be seen (cracks that produce slope deformation). These changes prepared the next undisturbed block of material (slab failure)/flowslides events as was confirmed during the research.

**Table 3:** Accumulated rain (mm) of flowslides events in the Arrupe slope (Figs. 21, 22, 23 and 24)

	Total for the event (mm)	Accumulated rain (mm)				
		24 h	48 h	15 days	25 days	30 days
04/09/2013	3	5.6	5.8	129.8	232.2	231.6
26/09/2013	4.4	0	0	61.4	121	150.6
25/05/2014	21.6	35.2	35.4	87	148.4	149
07/06/2014	25.4	0	0	229.6	309	321.6
18/06/2014	25.2	3.8	9.2	202.6	398	419

According to Sepulveda and Petley (2015) the number of fatal landsides is higher in Central America for moderate to low precipitations (50–150 mm/month). This information has a good correspondence to the threshold obtained for TBJ in this research.

Observing the slopes after the event, it appears that some of the flowslides that occur in TBJ could be categorized as “self-fluidization” and “secondary fluidization”. Fluidization can be distinguished from general sliding, which usually has an intact soil mass above the sliding surface (Ochiai et al. 2004). Moriwaki et al. (2004) defines “self-fluidization” when the landslide itself becomes fluid when slides from the source area. It needs high saturation of the soil or high water pore pressure that could involve complete or partial liquefaction. The “secondary fluidization” happens due the introduction of abundant water in streams in the slopes (Moriwaki et al. 2004). TMS3 picked a cycle of rapid increase of moisture content before failure or flowslide (Figs. 20, 21, 22, 23), connected with a particular rainfall and most of the flowslides happened in areas of preferential runoff so the “secondary fluidization” could predominate. These phenomena could be connected to the effects of subsurface removal of fine grained material (piping), the dilatation of the landslide mass in its preliminary stages of failure and soil porosity (Moriwaki et al. 2004). But to conclude more precisely about the detected field behavior, it might be necessary to install more equipment, use video camera to record the phenomenon and make full scale laboratory experiments (Ochiai et al. 2004; Moriwaki et al. 2004).

According to Savage and Baum (2010) the soils that suffer of compression when deformed reduce their pore space. This reduction in a saturated or nearly saturated soil produces an immediate increase in pore pressure, decreasing the soil strength and possibly resulting in a rapid evolution from soil slide to debris flow. Savage and Baum (2010) also concluded that an initial critical porosity of at least 20.5 % is required for rapid failure and debris flow fluidization in a sandy-loam soil (According to Rolo et al. 2004; Hernandez 2004 and own results, the average porosity of TBJ are 37.5–61.53 %). In TBJ most of the volume and travel distance of the flowslides are small (Values of H/L between 2.8 and 0.8 were common for small flows for TBJ pyroclastics) probably connected to the low fines (Fig. 10) content (Pierson 2010).

The use of numerical methods (e.g. SPH, FEM, FVM, MPM, CFD methods) to describe the flowslide behavior could help to simulate the phenomena (initiated by rains or an earthquake) and make hazards assessments. Authors like Huang et al. (2008), Medina et al. (2008), Huang et al. (2012a, b), Pastor et al. (2014) and Llano-Serna et al. (2015) had advances using some of the numerical methods. To continue this research is necessary to choose and test some of the methods and calibrate them according to real flowslides of the pyroclastic.

**Fig. 27:**
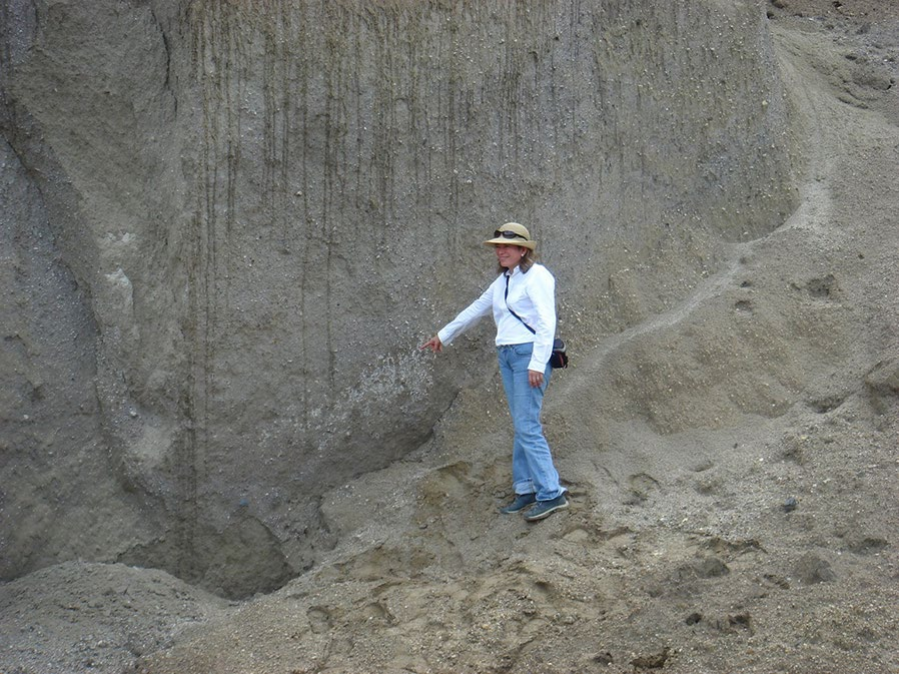
Sulfates coming from some sectors of TBJ slope in las Cañas River

It was noted during the monitoring time, that in spite to being close to saturation a good number of days (<5 kPa) some slopes (70°–90°) were stable (TBJ friction angle varies between 26° and 50°); this could be related to the effect of grain interlocking, cementation, surface crust, matric and osmotic suction. Eventually flowslides occurred after particular rainfalls. According to the monitoring results (Fig. 14, 15, 16), through all the year TBJ slopes undergo evapotranspiration or infiltration in the surface (this vary in each location). This suggested the possibility that the osmotic suction (and total suction) could be important as “ultimate apparent cohesion” that preserves the TBJ slopes stability when matric suction disappears. As mentioned before, a crust of raindrops compact the soil and minerals are formed in the face of TBJ slopes (Fig. 27), also BSCs could improve the stability at some level (Figs. 6, 26).

In order to understand the effect of the osmotic suction in the slopes by sulfates or salts, the total suction (matric + osmotic) was obtained in the laboratory using the filter paper technique (Fig. 11). Differences between the results obtained using the equations of total suction by Leong et al. (2002); Haghighi (2011) and Oliveira and Marinho (2006) are obvious, and an overestimation could be a problem. Leong et al. (2002) comprises two equations (matric and total); the other two use the same equation to obtain both suctions. According to Leong et al. (2002) the total suction calibration curve is not very sensitive to suctions less than 1000 kPa. For this reason it appears that the results closer to reality belongs to the Leong et al. (2002) equation. This indicates that more detail studies are necessary to confirm the importance of osmotic suction in TBJ; using equipment like the electrical conductivity of pore water extracted using pore fluid squeezer, psychrometers and the vapor equilibrium technique (Murray and Sivakumar 2010). The tensiometer and TMS3 are not capable to measure the osmotic suction underestimating its importance, but they can be helpful in building more criteria for an early warning system.

Results of conductivity made in various monitored areas and different TBJ units confirm that the pyroclastics are non-saline (2–16.2 μS/cm). pH values of the pyroclastics vary in 6.13–7.56 (Amaya and Hayem (2000) and own results). Also with ion chromatograph, the major inorganic cations and anions identified for UES G unit sample (with more occurrence) were Na^+^, Ca_2_^+^, SO_4_^2−^ and Cl^−^. In some areas of the slopes salts are visible but only as a crust and are not homogeneous through the entire surface (Fig. 27). According to Hernandez (2004) epsomite and gypsum are the main secondary minerals of the crust that forms in TBJ pyroclastics, acting as a weak cementation.

There are different positive and negative opinions, about the benefits of BSCs (Xiao et al. 2011; Jia et al. 2012; Kidron 2014; Xiao et al. 2014). In the monitored TBJ slopes an increasing in infiltration and water storage was recorded with the TMS3 (Figs. 14, 21). Also reducing of runoff and erosion was observed as well (Xiao et al. 2011). But in spite of the presence of BSCs in the surface of TBJ slopes, some flowslides were observed which could be connected to the high inclination of the slopes (Figs. 6, 26). During summer, the BSCs are in state of inactivity. Land management to preserve soil water conditions and its related problems could be improved with BSCs but more research is needed. Artificially grow moss-dominated biological soil crusts (moss crusts) can be also used to preserve disturbed areas (Xiao et al. 2011).

Several authors claim that the use of soil bioengineering using vegetation (roots) can improve the slope stability due to increasing suction, raindrop interception, reducing runoff and mechanical reinforcement (root anchorage) (Cazzuffi et al. 2006; Khalilnejad et al. 2012; Chirico et al. 2013). In spite of these benefits, during the fieldwork it was observed that a mechanical weathering associated to the root action causes fall of undisturbed block of material in the TBJ slopes faces (Fig. 6, 25, 26). Also the root area was the failure plane of flowslides. According to Comino and Druetta (2010) the roots contribution is not that effective in cohesionless soils. Also Zhang et al. (2014) said that the lignin and cellulose content of the roots and root diameter can control the tensile strength.

Bommer and Rodriguez (2002) said that if comparison is made between the 1965 earthquake (at the end of dry season) and the October 1986 quake (at the end of rainy season) located inside the Metropolitan Area of San Salvador (MASS) (Fig. 8). The area affected by landslides by the 1986 earthquake was five times larger with a higher number of landslides, despite it had a smaller magnitude. The 1986 earthquake triggered significant amount of flows, unlike the 1965 event.

Past events or the susceptibility to liquefaction of TBJ in its natural state, as landfill or alluvial sediments are reported by Schmidt-Thomé (1975); Lomnitz and Schulz (1966); Evans and Bent (2004); Jibson et al. (2004); Rolo et al. (2004). According to authors such Kramer (1996); Andrews and Martin (2000); Sassa and Wang (2010); Huang and Yu (2013) TBJ pyroclastics have some of the properties that fit the criteria for liquefaction of silty soils: geologic (Holocene), aerial deposit, affected by previous events, loose meta-stable granular soil, low content of clay (<10 %) and low liquid limit (<35). Liquefaction can be related to monotonic loading (e.g. an increase in load or sudden loss of toe support) or cyclic loading (e.g. artificial vibrations and earthquakes). According to Bommer and Rodriguez (2002) during earthquakes, tension cracks affect Tierra Blanca pyroclastics, initiating slides. Evans and Bent (2004) conclude that brittleness due to loss of apparent cohesion (suction) or generation of excess pore-water pressure in zones can produce flowslides during an earthquake.

As an unsaturated unconsolidated soil, the surface of TBJ slopes changes the moisture content constantly during the rainy season and near saturation state is usual during many days (Figs. 14, 15, 16, 20, 21); but also some units of TBJ (depending of its characteristics and situation) tend to store moisture for a longer time through most of the year. In this case is not necessary to have a shallow groundwater table to initiate liquefaction. Furthermore, there is a lot of perched groundwater linked to geology or broken waterlines.

## Conclusions

This research describe information of moisture content and suction with field monitoring and laboratory tests for a problematic unsaturated pyroclastic soil (TBJ) in the Metropolitan Area of San Salvador. The pyroclastic is affected by flowslides, debris/avalanche flows but also it can move like an undisturbed block of material. Being the flowslides more common but with a smaller volume, especially during the months between June and October. TMS3 from TOMST [time domain transmission (TDT)] and Quickdraw tensiometer were used in the field for regular monitoring. The tensiometer measures the matric suction directly, but it’s not able to measure suctions higher than 60–70 kPa. To use the TMS3, a calibration curve is necessary to convert the device reading to the gravimetric/volumetric water content. Only TMS3 was installed permanently in the field and some flowslides were recorded during the research time. It helped to understand changes in suction/moisture and its incidence in the behavior of the pyroclastics. TMS3 showed a good performance, are cheap and is generally independent of salinity and temperature allowing measurements over large spatial scales.

Rain intensity of the recorded flowslides in the pyroclastics (small volume) correspond with rains above 10 mm/h and for bigger flowslides with rains exceeding 58 mm/h rain. Gravimetric moisture content before flowslides were above 30 % and matric suction below 10 kPa. For the flowslides the threshold for initiating significant flowslides was above 20 mm for the whole event, greater than 87 mm for 15 days of accumulated rain and above 148 mm for 30 days of accumulated rain. More research is needed to have a representative data including other factors that can trigger the flowslides. It’s recommended to install more equipment for monitoring, use video-camera to record the phenomenon and make full scale laboratory experiments. The areas with subsurface groundwater flow and were the pyroclastics are saturated are more prone to be affected by liquefaction that could produce landslides. This condition needs to be studied in more detail.

The results show that not only climatic factors as temperature, rain and evaporation are important to build landslide criteria. Additionally information of suction–moisture content, seepage, weathering, topography, ground deformation, vibrations, cracks, vegetation/roots and the presence of crust covering the surface are necessary to increase the susceptibility to landslide. The presence of BSCs covering the slopes surface can protect somehow the steep slopes reducing the runoff process and mass wasting processes. For this reason is suggested the research of artificially grow moss-dominated biological soil crusts (moss crusts) that can be used to preserve disturbed areas.

The use of slope stabilization and land use measures that are environmental friendly are proposed with more emphasis. Examination for the use of the pyroclastics for geopolymer technology could be a good way to improve the pyroclastic behavior. Also the utilization of capillary barrier; a fine-grained soil layer placed over a coarse-grained soil layer can control the infiltration of rainwater into the slopes could be a possibility.
